# Effect of lipid saturation on amyloid-beta peptide partitioning and aggregation in neuronal membranes: molecular dynamics simulations

**DOI:** 10.1007/s00249-019-01407-x

**Published:** 2019-10-26

**Authors:** Nikolaos Ntarakas, Inna Ermilova, Alexander P. Lyubartsev

**Affiliations:** Department of Materials and Environmental Chemistry, Stockholm’s University, 10691 Stockholm, Sweden

**Keywords:** Amyloid-beta peptides, Molecular dynamics, Alzheimer disease, Polyunsaturated fatty acids, Lipid bilayers, Phospholipids, Omega-3

## Abstract

Aggregation of amyloid-$$\beta $$ (Aβ) peptides, cleaved from the amyloid precursor protein, is known as a precursor of the Alzheimer’s disease (AD). It is also known that Alzheimer’s disease is characterized by a substantial decrease of the amount of polyunsaturated lipids in the neuronal membranes of the frontal gray matter. To get insight into possible interconnection of these phenomena, we have carried out molecular dynamics simulations of two fragments of A$$\beta $$ peptide, A$$\beta $$$$_{1-28}$$ and A$$\beta $$$$_{26-40}$$, in four different lipid bilayers: two monocomponent ones (14:0-14:0 PC, 18:0-22:6 PC), and two bilayers containing mixtures of 18:0-18:0 PE, 22:6-22:6 PE, 16:0-16:0 PC and 18:1-18:1 PC lipids of composition mimicking neuronal membranes in a “healthy” and “AD” brain. The simulations showed that the presence of lipids with highly unsaturated 22:6*cis* fatty acids chains strongly affects the interaction of amyloid-$$\beta $$ peptides with lipid membranes. The polyunsaturated lipids cause stronger adsorption of A$$\beta $$-peptides by the membrane and lead to weaker binding between peptides when the latter form aggregates. This difference in the behaviour observed in monocomponent bilayers is propagated in a similar fashion to the mixed membranes mimicking composition of neuronal membranes in “healthy” and “AD” brains, with “healthy” membrane having higher fraction of polyunsaturated lipids. Our simulations give strong indication that it can be physical–chemical background of the interconnection between amyloid fibrillization causing Alzheimer’s disease, and content of polyunsaturated lipids in the neuronal membranes.

**Electronic supplementary material:**

The online version of this article (10.1007/s00249-019-01407-x) contains supplementary material, which is available to authorized users.

## Introduction

Alzheimer’s disease (AD) is a chronic neurodegenerative disease which is characterized by the aggregation of insoluble fibrillary amyloid-$\beta$ (A$\beta$) peptides in the extracellular space of neural tissues, resulting in neural atrophy (Khondker et al. 2017). A$\beta$ is a poly-residue peptide cleaved from the C-terminal region of the amyloid precursor protein (APP) (Rauk 2009). Different soluble A$\beta$ oligomers are considered to have a significant impact on the pathogenesis of the disease (Breydo et al. 2016). It is also known that AD disease is accompanied by specific changes of lipid composition in the neuronal brain membranes. Thus, AD brain is characterized by a substantial decrease of content of unsaturated lipids, and particularly lipids with docosahexaenoic (22:6*cis*) fatty acid chains (known also as $\omega -3$) in the frontal gray matter (Söderberg et al. 1991; Wood 2012). Regulation of 22:6*cis* fatty acids is also related to many other human diseases and afflictions (see review (Stillwell and Wassall 2003) and references therein). Lipids with 22:6*cis* fatty acid chains are present in neuronal membranes in substantially higher amount than in other tissues, however, their role in the membrane functioning remains largely unclear.

Full A$\beta _{1-42}$ peptide as well as its various fragments (for example, A$\beta _{1-28}$, A$\beta _{25-35}$, A$\beta _{26-40}$) have been extensively studied in the literature (Talafous et al. 1994; Kirshenbaum and Daggett 1995; Kowalik-Jankowska et al. 2003; Lau et al. 2006; Ionov et al. 2010; Rojas et al. 2011). The three-dimensional structure of A$\beta _{1-28}$ has been investigated with 2-dimensional nuclear magnetic resonance (NMR) and molecular dynamics (MD) (Talafous et al. 1994). It contains a bent $\alpha$-helix with the bend centred at VAL$_{12}$, and can produce both monomeric $\alpha$-helical and oligomeric $\beta$-sheet structures (Talafous et al. 1994). It is suggested in the literature that A$\beta _{1-28}$ forms fibrils with the cross-$\beta$ structure characteristic of the full-length A$\beta$ and takes up a helical conformation in solution (Rojas et al. 2011; Talafous et al. 1994; Kirshenbaum and Daggett 1995). The coordination properties of A$\beta _{1-28}$ have been determined by UV–Vis, CD and EPR techniques (Kowalik-Jankowska et al. 2003). A$\beta _{26-40}$ is less studied and only a few of its properties are known. The 26-40 fragment of A$\beta$ is strongly hydrophobic, the solubility of an isolated A$\beta _{26-40}$ is $6.2 \pm 1.2 \ \upmu$M (Han et al. 1995).

Besides numerous experimental works, several studies were made to model A$\beta$ peptides with simulations (Kirshenbaum and Daggett 1995; Ma and Nussinov 2002; Lee and Ham 2010; Zhao et al. 2011, 2018; Pannuzzo et al. 2013; Tran and Ha-Duong 2015; Rojas et al. 2011). Kirshenbaum and Daggett (1995) performed a molecular dynamics (MD) simulation of A$\beta _{1-28}$ to investigate the pH-dependent conformational behaviour of the peptide in aqueous solutions. Lee and Ham (2010) simulated the whole A$\beta _{1-42}$ with explicit water to examine misfolding, and they suggested an aggregation mechanism for the formation of fibrils. Another MD study found that the residues 23-28 could nucleate the folding of the A$\beta$ monomer and a bent in this region could be the rate-limiting step in A$\beta$ fibril formation (Tran and Ha-Duong 2015). Less common are simulations of A$\beta$ in lipid membranes. Lemkul and Bevan (2013) carried out simulations of a pair of A$\beta _{1-40}$ peptides in several saturated and monounsaturated membranes, paying attention on the conformational properties of the peptides and effect on the membrane structure. In subsequent study of the same laboratory (Brown and Bevan 2016), an A$\beta _{1-42}$ tetramer was simulated in a POPC membrane described by a united atom force field. Zhao et al. (2011) simulated three full-length A$\beta _{1-42}$ peptides preassembled in cholesterol-containing DPPC bilayer. Pannuzzo et al. (2013) studied A$\beta$(1-40) aggregation in POPC membrane by coarse-grained simulations. Davis and Berkowitz (2009a, b, 2010) have studied the behaviour of a single A$\beta (1-42)$ in several lipid bilayers using united atom force field. Lu et al. (2019) carried out simulations of an A$\beta$(29–42) dimer in several membranes including polyunsaturated 18:0/20:4(*n*−6) PC and 18:0/22:6(*n*−3) PC bilayers. We are, however, not aware of any fully atomistic simulations of aggregation of A$\beta$ peptides in lipid membranes with a systematic analysis of the role of lipid unsaturation.

To reveal possible interconnection between the aggregation behaviour of the A$\beta$ peptides, their interaction with neuronal membranes, and membrane composition, we have carried our molecular dynamics simulations of two types of $\beta$-amyloid peptides in several bilayers with varying content of polyunsaturated lipids. The peptides, A$\beta (1-28)$ and A$\beta (26-40)$, were taken from two different parts of the A$\beta (1-42)$ sequence, and particular sequences were selected due to previous studies of these peptides available in the literature (Kobayashi et al. 1994; Ionov et al. 2010; Yates et al. 2013; Xiong et al. 2014; Barrett et al. 2016; Xiong and JiJi 2017).

## Models and methods

In this work, we present results of two series of microsecond-long simulations of A$\beta _{1-28}$ and A$\beta _{26-40}$ peptides in lipid membranes (see peptide sequences in Table 1). In the first one, behaviour of the peptides was studied in monocomponent bilayers composed of either fully saturated 14:0-14:0 PC (1,2-dimyristoyl-sn-glycero-3-phosphocholine, or DMPC), or strongly unsaturated 18:0-22:6 PC (1-stearoyl-2-docosahexaenoyl-sn-glycero-3-phosphocholine, or SDPC) lipids. In the second one, two bilayers composed of mixtures of 18:0-18:0 PE (1,2-Distearoyl-sn-glycero-3-phosphoethanolamine, or DSPE), 22:6-22:6 PE (1,2-didocosahexaenoyl-sn-glycero-3-phosphoethanolamine, or DDPE), 16:0-16:0 PC (1,2-dipalmitoyl-sn-glycero-3-phosphocholine, or DPPC) and 18:1-18:1 PC (1,2-Dioleoyl-sn-glycero-3-phosphocholine, or DOPC) lipids were investigated in the presence of A$\beta _{1-28}$ and/or A$\beta _{26-40}$ peptides. The number of lipids in the two mixed bilayers were chosen proportionally to the fraction of these lipids in the frontal gray matter of healthy and AD brains, as it was determined by Söderberg et al. (1991, 1992). According to that study, the fraction of polyunsaturated 22:6 fatty acid chains decreases from 23% in a healthy brain to 12% in an AD brain, the fraction of saturated lipids is increasing from 45 to 57% while the fraction of monounsaturated chains shows a small increase by 2% in an AD brain. The work by Söderberg et al. (1991) provided only an overall composition of fatty acids without explicit breakdown over specific lipids, still it unambiguously shows that the most abundant fatty acids chains (lipid tails) are 16:0, 18:0, 18:1(n-9), and 22:6(n-3), while lipid headgroups are mostly PC or PE. We thereafter have chosen four lipid types having symmetric tails of each of these four types, two of them representing PC lipids and two other PE lipids. The number of molecules of each type in each of these simulations is given in Tables 2 and 3, and it reflects the conclusion of work (Söderberg et al. 1991) that membranes of a healthy brain contain a higher fraction of polyunsaturated lipids compared to those of an AD brain. The two mixed bilayer systems are called “Normal” and “AD” through the text.

**Table 1:** Amino acid sequence of the full peptide and the chosen fragments

Peptide fragment	Amino acid sequence
A$\beta _{1-42}$	DAEFRHDSGYEVHHQKLVFFAEDVGSNKGAIIGLMVGGVVIA
A$\beta _{1-28}$	DAEFRHDSGYEVHHQKLVFFAEDVGSNK
A$\beta _{26-40}$	SNKGAIIGLMVGGVV

**Table 2:** Composition of monocomponent bilayers: number of molecules in each system

Type	Saturated		Polyunsaturated	
System	1	2	3	4
14:0-14:0 PC (DMPC)	200	200	0	0
18:0-22:6 PC (SDPC)	0	0	200	200
A$\beta _{1-28}$	4	0	4	0
A$\beta _{26-40}$	0	9	0	9
Water	20,000	20,000	20,000	20,000

**Table 3:** Composition of mixed bilayers: number of molecules in each system

Type		Normal			AD	
System	5	6	7	8	9	10
18:0-18:0 PE (DSPE)	60	60	60	77	77	77
22:6-22:6 PE (DDPE)	40	40	40	23	23	23
16:0-16:0 PC (DPPC)	65	65	65	68	68	68
18:1-18:1 PC (DOPC)	35	35	35	32	32	32
A$\beta _{1-28}$	4	0	2	4	0	2
A$\beta _{26-40}$	0	9	4	0	9	4
Water	20,000	20,000	20,000	20,000	20,000	20,000

Lipids were described by the Slipids force field (Jämbeck and Lyubartsev 2012a, b) which is well established force field for simulations of lipid bilayers (Lyubartsev and Rabinovich 2016) including also polyunsaturated lipids (Ermilova and Lyubartsev 2016). The force field parameters, as well as molecular topology files in the Gromacs format (Hess et al. 2008), are available from Slipids v.2.0 archive at Zenodo repository (Jämbeck et al. 2016). The peptides were presented by the General Amber Force Field (GAFF) (Wang et al. 2004). Recently, several comparative studies of different force fields to describe peptide structure have been carried out (Gerben et al. 2014; Somavarapu and Kepp 2016; Carballo-Pacheco and Strodel 2017; Robustelli et al. 2018) but their results on the force field performance were not unambiguous. GAFF, derived on the basis of Amber99 parameter set, was designed to provide better transferability of parameters over a wide range of organic molecules, which can be an advantage for description of intrinsically disordered peptides such as A$\beta$. Furthermore, GAFF showed the best performance to describe partitioning of solute molecules across bilayer with lipids described by the Slipids force field (Paloncýová et al. 2014). Previously, GAFF was used in simulations of peptides in a number of works (Tan et al. 2010; Zhu et al. 2016; Xu et al. 2016; Kang et al. 2016).

The initial peptide structures were prepared by the Avogadro molecular editor (Hanwell et al. 2012) with default parameters for $\phi$ and $\psi$ torsion angles (at 180$^\circ$ providing a stretched peptide conformation) and then processed by the *acpype* utility (Sousa da Silva and Vranken 2012) to produce molecular topology files in the Gromacs format. Ionizable residues were taken in the neutral form which can be justified that in the membrane interior, as well as in crowded environments, pKa of amino acids is shifted towards the neutral forms (Isom et al. 2008; Teixeira et al. 2016). Peptides were optimized and equilibrated in water environment for 50 ns to produce starting configurations for simulations with bilayers. In the process of optimization, the peptides rapidly adopted compact conformations with predominant $\alpha$-helical structure (see starting snapshots in Fig. S1 of the Supporting Information). Water was represented by the TIP3P model (Jorgensen et al. 1983).

The monocomponent lipid bilayers containing 14:0-14:0 PC and 18:0-22:6 PC and water were built using standard routines of the GROMACS-4.6.7 software (Hess et al. 2008). The mixed bilayers were created with lipid compositions mimicking the contents of gray matter according to (Söderberg et al. 1991), see Table  3. The initial structures of lipids were prepared stretched along *Z* direction, and then assembled into the bilayer structure using in-house tools. The prepared bilayers were hydrated by water and then simulated without peptides in semianisotropic NPT-ensemble for 100 ns. After the equilibration of each lipid bilayer, water molecules were taken away from the simulation boxes and peptides, optimized in a water environment, were inserted outside the membrane well separated from each other (using “insert-molecules” utility of Gromacs). Then water was added again by Gromacs utility “solvate”. Examples of the starting configurations for the three types of simulated systems are shown in Fig. S1 of the Supporting Information. The systems, containing lipid bilayers, peptides and water were further pre-equilibrated again for 100 ns under pressure 1 atm and temperature 303 K, providing starting configuration for the 1 $\upmu$s production simulations. The sizes of equilibrated systems were approximately 8 nm $\times$ 8 nm $\times$ 19 nm in *x*, *y*, *z* directions, respectively.

The molecular dynamics simulations were run with the GROMACS-4.6.7 software (Hess et al. 2008). All covalent bonds were constrained by the LINCS algorithm (Hess 2008). The NPT-ensemble was run at 1.013 bar pressure which was kept constant by the semianisotropic Berendsen barostat (Berendsen et al. 1984), with separate pressure control in *z* direction and in the *xy*-plane. The temperature was controlled by the V-Rescale thermostat (Bussi et al. 2007). The leap-frog algorithm (Berendsen et al. 1995) with time step 2 fs and a Verlet cutoff scheme (Sciacca et al. 2016) with cutoff radius 1 nm were used for integration equation of motion. Electrostatic forces were treated by the particle-mesh Ewald summation method (PME) (Darden et al. 1993). The total simulation time for each system was 1 $\upmu$s ($5 \times 10^8$ MD steps) of which the last 500 ns were used for computation of the properties of interest. Snapshots of the final configurations of the simulated systems are given in Figs. S2–S4 of the Supporting Information. The analysis of the results was performed using utilities from the MDynaMix simulation package (Lyubartsev and Laaksonen 2000).

**Fig. 1:**
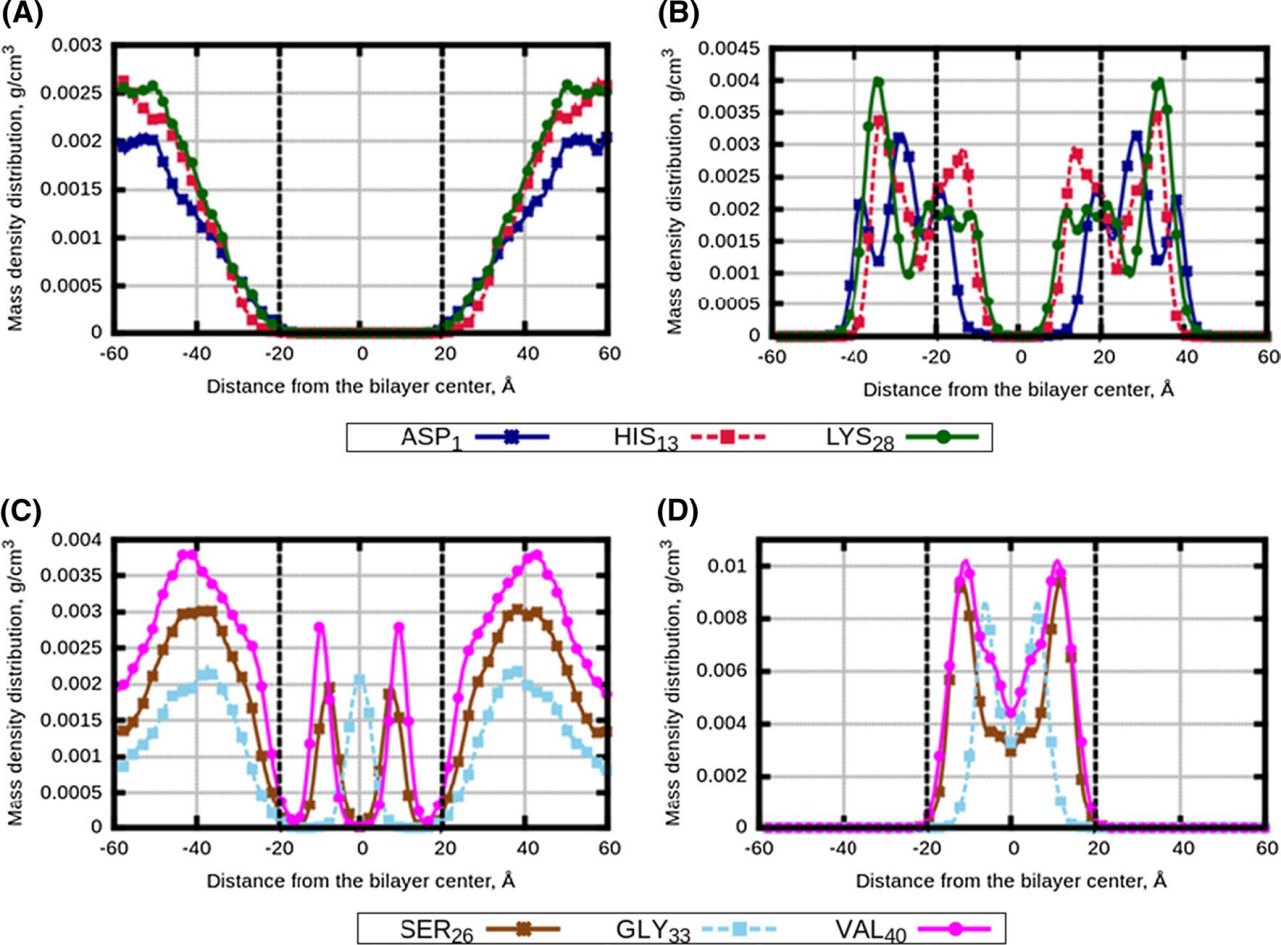
Density distributions of selected amino acids of A$\beta$ peptides: **a** 14:0-14:0 PC bilayer with A$\beta _{1-28}$ (System 5), **b** 18:0-22:6 PC bilayer with A$\beta _{1-28}$ (System 3), **c** 14:0-14:0 PC bilayer with A$\beta _{26-40}$ (System 2) and **d** 18:0-22:6 bilayer with A$\beta _{26-40}$ (System 4). For each peptide three amino acids were taken from the beginning, middle and end of the peptide sequence: ASP$_1$, HIS$_{13}$ and LYS$_{26}$ of A$\beta _{1-28}$, and SER$_{26}$, GLY$_{33}$ and VAL$_{40}$ residues of A$\beta _{26-40}$. Vertical black dotted lines are $PO_{4}$ groups of lipid heads

**Fig. 2:**
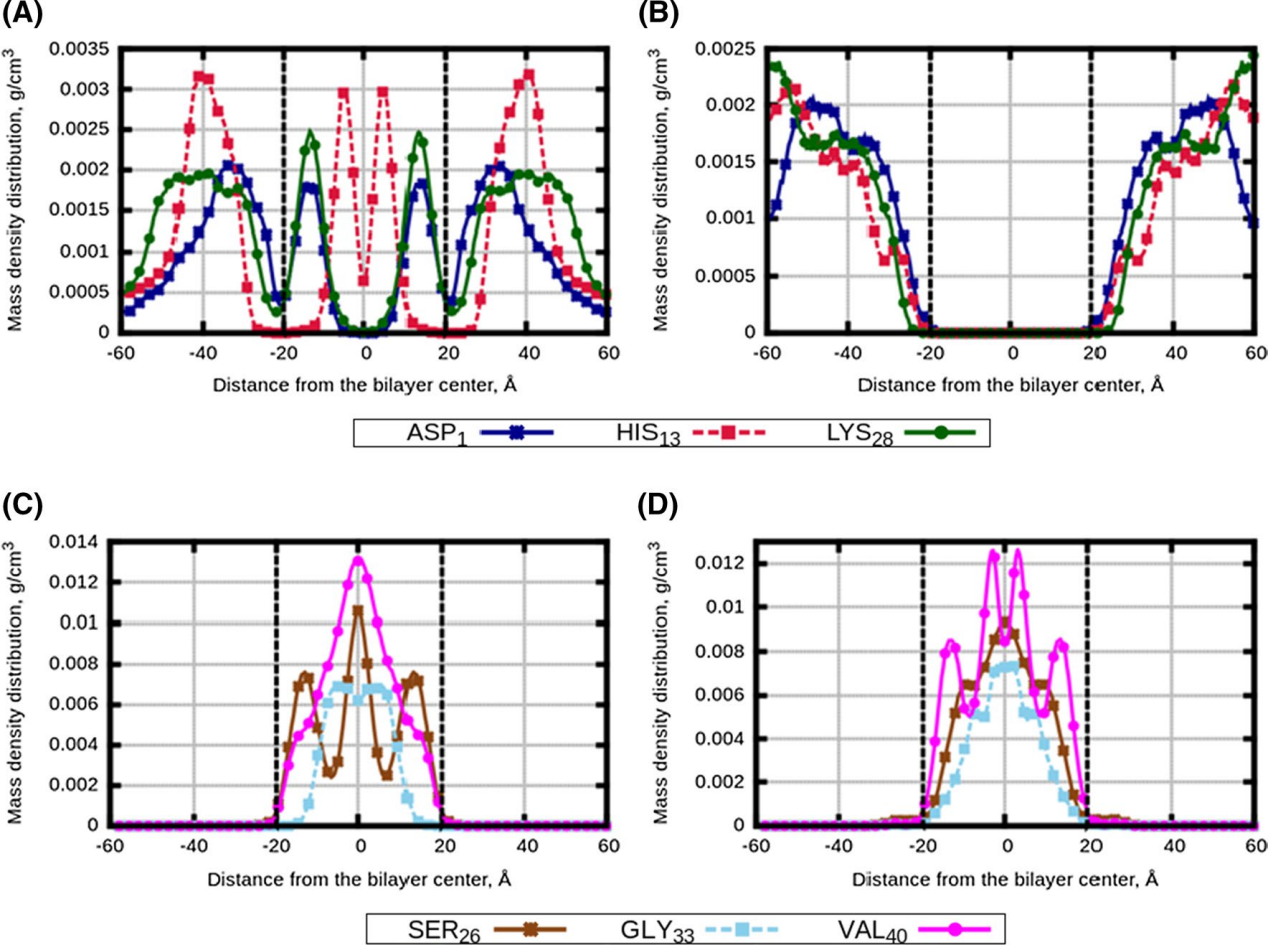
Density distributions of selected amino acids of A$\beta$ peptides in mixed bilayers: **a** “Normal” bilayer with A$\beta _{1-28}$ (System 5), **b** “AD” bilayer with A$\beta _{1-28}$ (System 8), **c** “Normal” bilayer with A$\beta _{26-40}$ (System 6) and **D** “AD” bilayer with A$\beta _{26-40}$ (System 9). Vertical black dotted lines are $PO_{4}$ groups of lipid heads

## Results and discussion

### Peptides distribution in bilayers

To characterize localization of peptides relative to the membrane and their distribution, we computed mass density profiles (density distributions) of three amino acids taken from the beginning, middle and end of each peptide, which are ASP$_1$, HIS$_{13}$ and LYS$_{28}$ residues of A$\beta _{1-28}$, and SER$_{26}$, GLY$_{33}$ and VAL$_{40}$ residues of A$\beta _{26-40}$. The profiles for monocomponent bilayers, symmetrized relative to the bilayer midplane, are shown in Fig. 1. One can see that both peptides have a stronger preference to be associated with the polyunsaturated membrane compared to the saturated one. The A$\beta _{1-28}$ peptide stays out of the 14:0-14:0 PC membrane (Fig. 1a), while in the 18:0-22.6 PC membrane it prefers to be at the membrane surface (Fig. 1b) partially entering the membrane. The A$\beta _{26-40}$ peptide stays mostly outside the 14:0-14:0 PC membrane, though certain molecules can go inside the membrane which is reflected by the local maxima of the density in the membrane interior. However, in the case of polyunsaturated 18:0-22:6 PC membrane, all A$\beta _{26-40}$ peptides reside inside the bilayer. Figures S5–S16 of the Supporting Information show peptides density distributions obtained during different time intervals of the simulations. One can see that the noted trends in the peptide behaviour appear already in the initial (equilibration) stage of the simulations during 100–200 ns, and become stable during the second half of the simulations. This also confirms that the distributions obtained by averaging over the second part of the simulations and shown in the main text, correspond to equilibrated positions of the peptides relative to the membrane.

**Fig. 3:**
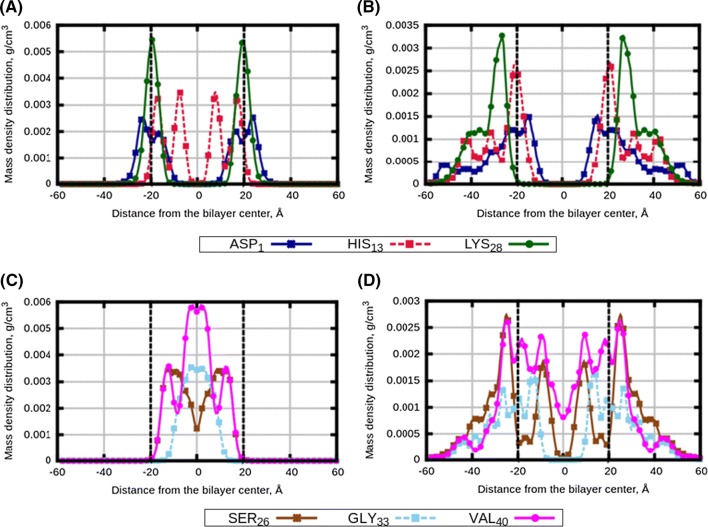
Density distribution profiles of selected amino acids of A$\beta$ peptides: **a**, **c** “Normal” membrane with two types of peptides (System 7); **b**, **d** “AD” membrane with two types of peptides (System 10); **a**, **b** amino acids of A$\beta _{1-28}$ peptides; **c**, **d** amino acids of A$\beta _{26-40}$ peptides. Vertical black dotted lines are $PO_{4}$ groups of lipid heads

Analysis of the peptide distributions in the mixed membranes shows similar trends. Here, we carried out simulations with each of peptides taken separately (Systems 5,6,8,9), as well as with mixture of A$\beta _{1-28}$ and A$\beta _{26-40}$ peptides (Systems 7 and 10, see definitions in Table 3). A$\beta _{1-28}$ can penetrate “Normal” membrane with high content of polyunsaturated lipids, while it does not penetrate into “AD” membrane (Fig. 2a, b). Behaviour of A$\beta _{26-40}$ is similar in the both membranes, where the peptides are found inside membrane in the both cases (Fig. 2c, d). Still, somewhat broader distribution can be inferred in case of “AD” membrane, mostly noticeable for the ending part of peptide given by the residue VAL$_{40}$.

In Fig. 3, we show density distribution from simulations when both types of peptides were present in “Normal” and “AD” membranes. In both bilayers, distribution of A$\beta _{1-28}$ turned out to be shifted to membrane surface (compared to the case when A$\beta _{1-28}$ was the only peptide in the simulation, Fig. 2), while distribution of A$\beta _{26-40}$ becomes partially spread outside the “AD” membrane compared to the case when A$\beta _{26-40}$ was the only peptide. Clearly, the two type of peptides affect distribution of each other by building common clusters, as it also seen in the snapshots (Fig. S4 of the Supporting Information).

Non-symmetrized density distributions of the peptides are also shown in Figs. S17–S19 of the Supporting Information. One can see that in cases when peptides aggregate in the water phase and not enter the membrane, they typically stay at the same membrane side. However, in cases when peptides enter the membrane, their distributions appear to be more symmetric relative to the membrane middle plane.

**Fig. 4:**
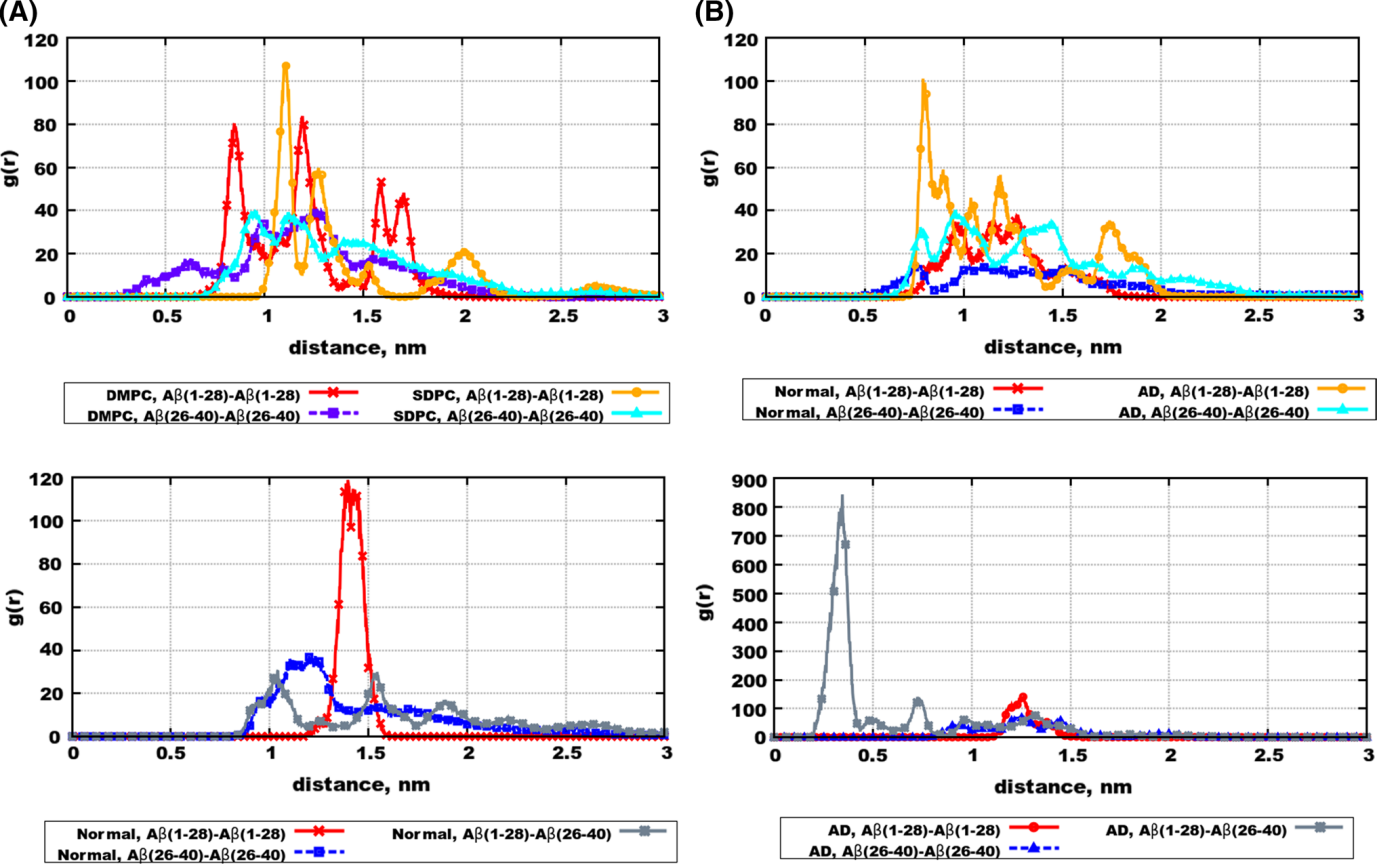
Intermolecular radial distribution functions between centres of mass of peptides in: **a** monocomponent bilayers (Systems 1,2,3,4), **b** mixed bilayers with one type of peptides (Systems 5,6,8,9), **c** “Normal” membrane with two peptide types (System 7), **d** “AD” membrane with two peptide types (System 10)

We can further comment that results of our simulations of A$\beta (1-28)$ and A$\beta (26-40)$ in 14:0-14:0 PC lipid bilayer are consistent with experimental findings of Ionov et al. (2010) where it was found that A$\beta (1-28)$ was aggregating on the surface of DMPC/DPPG membrane while A$\beta (25-40)$ was inserted in the bilayer. Also, Yip and McLaurin (2001) have shown that A$\beta (1-28)$ was aggregating on the surfaces of brain lipid membrane as well as on lipid bilayer containing DMPC that is also in agreement with our simulations of both pure 14:0-14:0 PC and mixed membranes.

### Peptide aggregation

Quantitatively, interaction of peptides between each other, and clusters formation can be characterized by radial distribution functions (RDF). RDFs between centre of masses of peptides are shown in Fig. 4. In all cases, RDFs reach high values at 10–15 Å manifesting clustering behaviour. At the same time, there are certain differences between the two types of peptides, as well as between different bilayers. Generally, A$\beta _{26-40}$–A$\beta _{26-40}$ RDFs are smoother than A$\beta _{1-28}$- A$\beta _{1-28}$ RDFs, which means that A$\beta _{1-28}$ clusters are more structured. Furthermore, one can note that in case of pure bilayers (Fig. 4a) RDFs of both peptides have higher values at lower distance in case of saturated 14:0-14:0 PC bilayer, indicating stronger binding into clusters. In case of mixed bilayers, stronger clustering (larger RDF at smaller distances) can be observed for “AD” bilayer which has higher fraction of saturated lipids. This conclusion can be also illustrated by snapshots in Fig. S3 of the supporting information, where peptides form a single cluster in case of “AD” bilayer (Fig. S3B, D) while they are more dispersed in case of “Normal” bilayer (Fig. S3A, C).

Figure 4c, d shows RDFs between polypeptide centre of masses in case when both types of peptides are present. Again, one can see that stronger clustering is observed for the “AD” bilayer. Furthermore, in this bilayer the RDF between different peptides (A$\beta _{1-28}$–A$\beta _{26-40}$) is substantially stronger than the RDF between likewise peptides. The two peptides taken together can be seen almost as a single full-length peptide A$\beta _{1-40}$, so this result can be interpreted as saturated lipids (or AD membrane) favour aggregation of the full A$\beta _{1-40}$ peptides. The strong interaction between A$\beta _{1-28}$ and A$\beta _{26-40}$ explains also why density distribution of A$\beta _{26-40}$ spreads outside the membrane in case of “AD” membrane (Fig. 2d) while A$\beta _{26-40}$ is fully adsorbed in membrane in case where there are no A$\beta _{1-28}$ (Fig. 2c).

In addition to the RDF analysis, we have computed contact maps of peptide residues through the whole simulations. The results are presented in Figs. S20–S29 of the Supporting Information. In each case, the simulated trajectory was divided into five 200 ns fragments, and results averaged within each fragment are shown on separate panels. On all the maps one can see frequent contacts of residues separated by four amino acids along the chain, which assumes typically $\alpha$-helical peptide conformations. Intermolecular peptide contacts can be also seen on all the maps through the simulations which means that different peptides are often in contact with each other. The contact maxima are changing, however, through the simulations, thus the peptide aggregates do not have stable structure but individual peptides change the neighbours on a hundred-nanosecond time scale. One can also see a trend of having less intermolecular peptide-peptide contacts in case of polyunsaturated bilayers (pure 18:0-22:6 PC and “Normal” mixed bilayer) which is consistent with conclusions made from the RDF calculations that polyunsaturated lipids relax aggregation of A$\beta$ peptides.

We have also determined peptides secondary structures and their changes through the simulations. The results are shown in Figs. S30–S40 of the Supporting information. The prevailing secondary structure is “turn” corresponding to $\alpha$-helical structure, which is also consistent with the analysis of contact maps, and with the systems snapshots. The prevailing $\alpha$-helical structure of aggregated A$\beta$ peptides was also observed in atomistic simulations of Pannuzzo et al. (2013). Less frequent are 3-10 helix structures (seen mostly in A$\beta _{1-28}$ peptides) and “bridge” appearing in A$\beta _{26-40}$ peptides. A$\beta _{26-40}$ peptides have also a higher fraction of unstructured (free) conformations. The peptide secondary structure is dynamic, it is different between different peptide molecules at each time moment, and it is changing during the simulations.

### Effect of peptides on the bilayer structure

Since in all considered cases the peptides interact with the bilayers, either by clustering at the surface or entering inside the membrane, it is instructive to investigate which effect the peptides exert on the bilayer structure. For this purpose, we have computed average area per lipid in the simulated systems and NMR order parameters, and compared with results of simulations of the corresponding bilayers without peptides. For pure monocomponent 14:0-14:0 PC and 18:0-22:6 PC bilayers we used data from previous works (Jämbeck and Lyubartsev 2012a; Ermilova and Lyubartsev 2016), while for the mixed bilayers the areas and order parameters were determined from additional 100 ns simulations of the pre-equilibrated bilayers without peptides (see section Model and Methods).

Computed values of the average areas per lipid are shown in Table 4. In all cases, the presence of peptides leads to a small, typically within 2–3 $\AA ^2$ increase of the area. One can note that the increase is somewhat larger in cases when peptides are entering the membrane, but this difference is within the computational error limits. This means that even in cases when peptides are bound on the membrane surface remaining in the water phase, they still affect the bilayer structure due to interactions with lipid headgroups.

**Table 4:** Average areas per lipid

Bilayer	Area per lipid, Å$^{2}$	Error, Å$^{2}$
14:0-14:0 PC (pure) (Jämbeck and Lyubartsev 2012a)	60.8	0.5
14:0-14:0 PC & A$\beta (1-28)$	63.0	0.5
14:0-14:0 PC & A$\beta (26-40)$	63.2	0.5
18:0-22:6 PC (pure) (Ermilova and Lyubartsev 2016)	68.6	0.7
18:0-22:6 PC & A$\beta (1-28)$	71.4	0.9
18:0-22:6 PC & A$\beta (26-40)$	71.5	0.7
“Normal” (pure)	60.6	0.5
“Normal” & A$\beta (1-28)$	62.	0.5
“Normal” & A$\beta (26-40)$	63.3	0.6
“Normal” & A$\beta (1-28)$ & A$\beta (26-40)$	63.7	0.5
“AD” (pure)	57.4	0.8
“AD” & A$\beta (1-28)$	57.8	1.1
“AD” & A$\beta (26-40)$	59.9	1.2
“AD” & A$\beta (1-28)$ & A$\beta (26-40)$	60.2	1.7

**Fig. 5:**
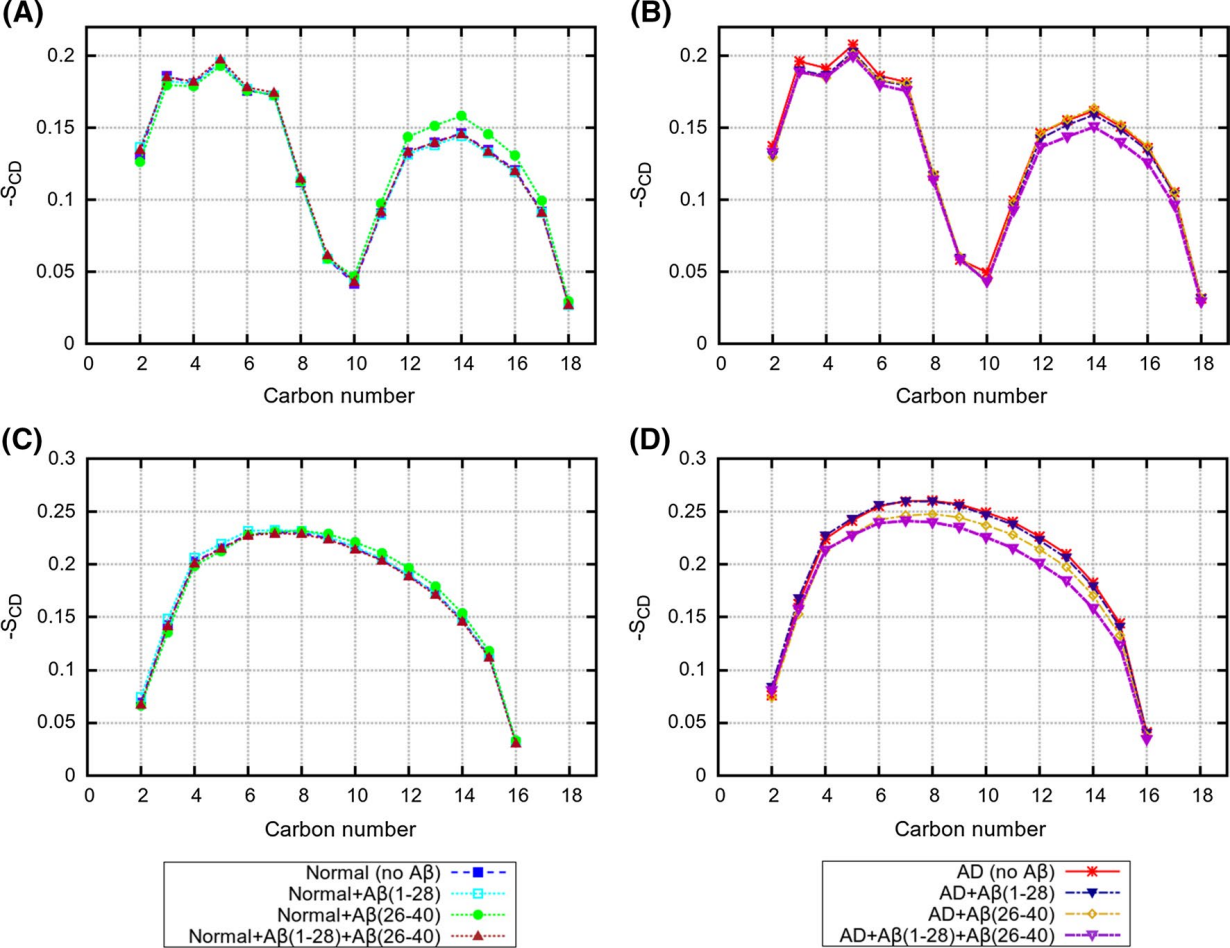
Deuterium order parameters for $sn-1$ lipid tails for mixed systems with and without peptides. **a** 18:1-18:1 PC in “Normal” bilayer; **b** 18:1-18:1 PC in “AD” bilayer; **c** 16:0-16:0 PC in “Normal” bilayer; **d** 16:0-16:0 PC in “AD” bilayer

**Fig. 6:**
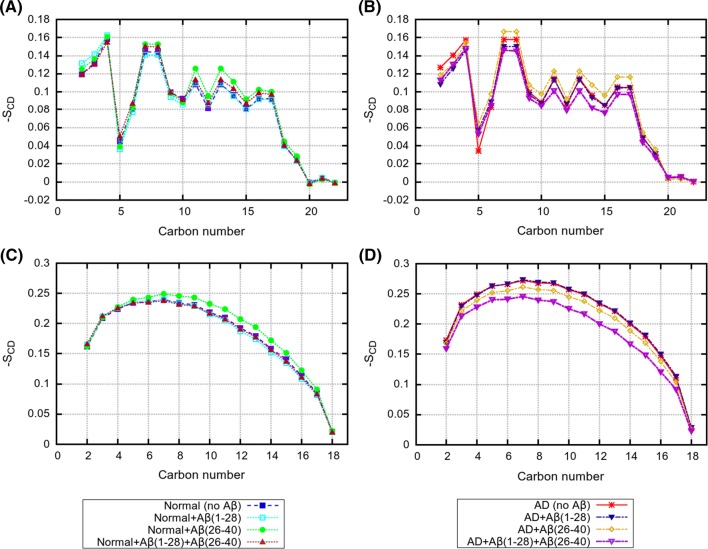
Deuterium order parameters for $sn-1$ lipid tails for mixed systems with and without peptides. **a** 22:6-22:6 PE in “Normal” bilayer; **b** 22:6-22:6 PE in “AD” bilayer; **c** 18:0-18:0 PE in “Normal” bilayer; **d** 18:0-18:0 PE in “AD” bilayer

The deuterium order parameters of the CH bonds in lipid tails, for each lipid type in “Normal” and “AD” bilayers, are shown in Figs. 5, 6. One can see that for each lipid type, the order parameter profile has typical features seen in many previous works (Jämbeck and Lyubartsev 2012a, a; Ermilova and Lyubartsev 2016). Thus, for fully saturated 16:0-16:0 PC and 18:0-18:0 PC lipids the order parameters are increasing reaching plateau of magnitude 0.2–0.25 at 4–10 carbons and then going down to zero at the end of tail; monounsaturated 18:1-18:1 PC lipids have a strong drop in the order parameter at the position of the double bond, while polyunsaturated 22:6-22:6 PC lipids show a zig-zag like profile with substantially lower values of the order parameters.

Our results on the average lipid areas and order parameters show that the presence of peptides have only a small effect on the structure of lipid bilayer, with somewhat larger effect for the “AD” bilayer. It is likely that the presence of strongly unsaturated 22:6-22:6 PE lipids with highly flexible tails which can easily change their conformation relax mechanical stress introduced by the peptides when the later entering the membrane. It is also plausible that higher flexibility of 22:6*cis* chains and lower order introduced by them into bilayer is the driving force which favour insertion of peptides into bilayers rich by this type of chains. Previously, Pannuzzo et al. (2013) proposed, on the basis of coarse-grained simulations, that aggregation of peptides in membranes may cause mechanical stress and induce membrane curvature. Pore formation in membrane induced by A$\beta$ peptides was also discussed (Scala et al. 2016) as a possible toxicity mechanism, and it was demonstrated that the presence of considerable amount of cholesterol was needed for that.

## Conclusions

Summarizing results presented here, we can conclude that the presence of lipids with highly unsaturated 22:6-*cis* chains strongly affects the interaction of amyloid-$\beta$ peptides with lipid membranes. Our simulations unambiguously showed that polyunsaturated lipids cause stronger adsorption of A$\beta$-peptides by the membrane and lead to weaker binding between peptides when the later form aggregates. The difference in the behaviour observed in the monocomponent bilayers is propagated in a similar fashion to the mixed membranes mimicking composition of healthy and AD brains, with healthy membrane having higher fraction of unsaturated lipids. It was demonstrated previously in in vitro studies (Hossain et al. 2009), that docosahexaenoic acid (22:6(*n*−3)chain) inhibits A$\beta (1-42)$ fibril formation, thus adsorption of A$\beta$-peptides into membrane rich by polyunsaturated lipids would suppress formation of A$\beta$ aggregates on the membrane surface. Also, Electronic Spin Resonance (ESR) experiments (Vitiello et al. 2013) showed that 22:6*cis* PC lipids enhance A$\beta _{25-35}$ peptide interaction with lipid membrane, favouring a deeper internalization of the peptides among the lipid acyl chains, and inhibiting release of the peptide from membrane with subsequent fibrillization. Our simulations, carried for two other A$\beta$-peptides, fully support this picture. One can further speculate that by interacting with A$\beta$-peptides, polyunsaturated lipids divert the peptides from the cholesterol-rich raft domains which are known as promoters of the amyloid aggregation, and enhance clearance of A$\beta$-peptides due to interaction with A$\beta$-degrading enzymes (Boudrault et al. 2009; Grimm et al. 2017). Overall, our simulations give strong indication that interconnection between amyloid aggregation causing AD disease, and content of polyunsaturated lipids in neuronal membranes has a molecular, physical–chemical background.

## Electronic supplementary material

Below is the link to the electronic supplementary material.

Supplementary material 1 (pdf 6157 KB)
