# Supplementary material for: Effect of lipid saturation on amyloid-beta peptide partitioning and aggregation in neuronal membranes: molecular dynamics simulations
Source: Eur Biophys J. 2019 Oct 26;48(8):813–24. doi: 10.1007/s00249-019-01407-x (PMC6853862; doi:10.1007/s00249-019-01407-x)
Supplement: Supplementary file 1 — Supplementary material 1 (pdf 6157 KB) [file 249_2019_1407_MOESM1_ESM.pdf]

Effect of lipid saturation on amyloid-beta peptides partitioning and aggregation in neuronal membranes: Molecular dynamics simulations. Supporting Information

Nikolaos Ntarakas, Inna Ermilova, and Alexander P. Lyubartsev*

Department of Materials and Environmental Chemistry, Stockholm University, SE 106 91, Stockholm, Sweden

tel. +46-8161193

E-mail: alexander.lyubartsev@mmk.su.se

Content

⊛ Snapshots

Figure S1: Snapshots of starting configurations

Figure S2: Snapshots of monocomponent bilayers

Figure S3: Snapshots of mixed bilayers with one type of peptides

Figure S4: Snapshots of mixed bilayers with two types of peptides

⊛ Convergence of the density profiles

Figures S5-S8: Plots of mass density profiles for 3 selected aminoacids for simulations of monocomponent bilayers (14:0-14:0 PC and 18:0-22:6 PC, runs 1 - 4)

Figures S9-S12: Plots of mass density profiles for 3 selected aminoacids for simulations of mixed "normal" and "AD" membranes with one type of peptides (runs 5,6,8,9).

Figures S13-S16: Plots of mass density profiles for 3 selected aminoacids for simulations of "normal" and "AD" membranes with two types of peptides (runs 7,10).

Figures S17-S19: Plots of non-symmetrized mass density profiles for peptides in different simulations.

⊛ Contact maps through the whole simulation time

Figures S20-S29: Contact maps of peptides for the simulated systems, calculated during five time intervals of the simulations.

⊛ Secondary structures of peptides

Figures S30-S40: Secondary structures of peptides through the whole simulations.

Snapshots

Snapshots illustrating initial state of the three types of simulated systems (Fig. S1), and snapshots of the simulated systems in the end of simulations, Figs. S2-S4. All snapshots were generated by the VMD software.[1]

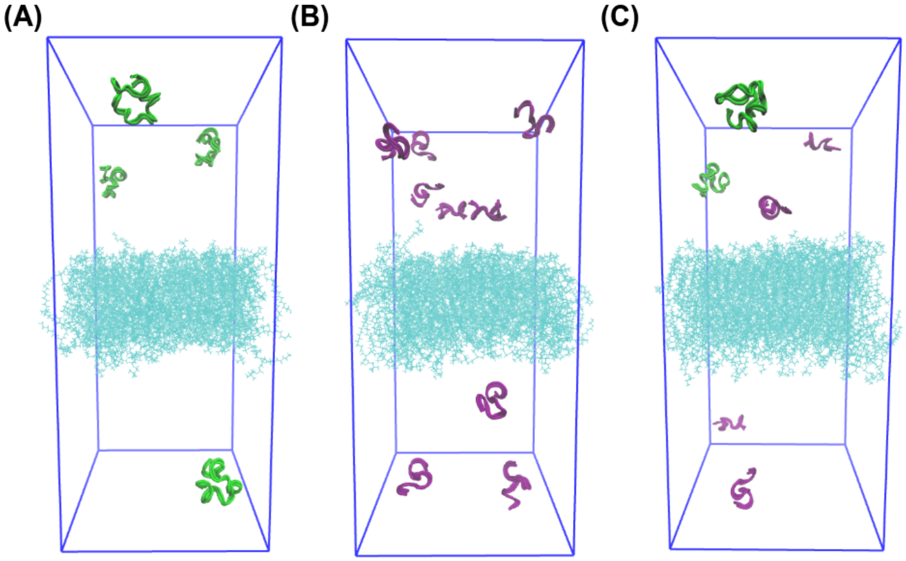

Figure S1: Starting configurations for 3 types of systems. (A) Systems with $A\beta(1-28)$ (B) Systems with $A\beta(26-40)$ (C) Systems with both $A\beta(1-28)$ and $A\beta(26-40)$. A$\beta(1-28)$ peptides are represented by green ribbons. A$\beta(26-40)$ peptides are represented by purple ribbons. Lipid bilayers are cyan molecules in the middle of simulation boxes.

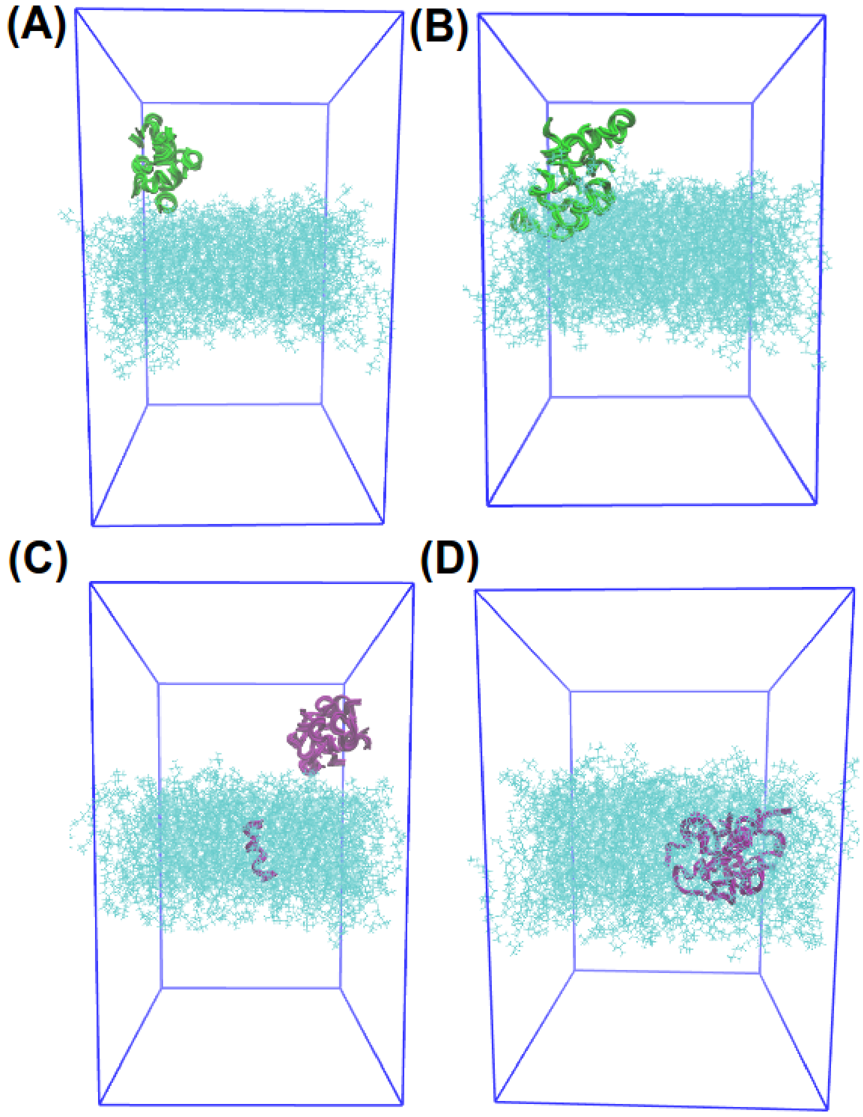

Figure S2: Snapshots of monocomponent bilayers in the end of simulations: (A) DMPC with Aβ_{1-28} (System 1), (B) SDPC with Aβ_{1-28} (System 3), (C) DMPC with Aβ_{26-40} (System 2), (D) SDPC with Aβ_{26-40} (System 4, notations of systems is given in Table 1 of the main text). PC lipids are represented as blue lines for bonds, peptides are shown as ribbons, Aβ_{1-28} is in green, and Aβ_{26-40} is in purple.

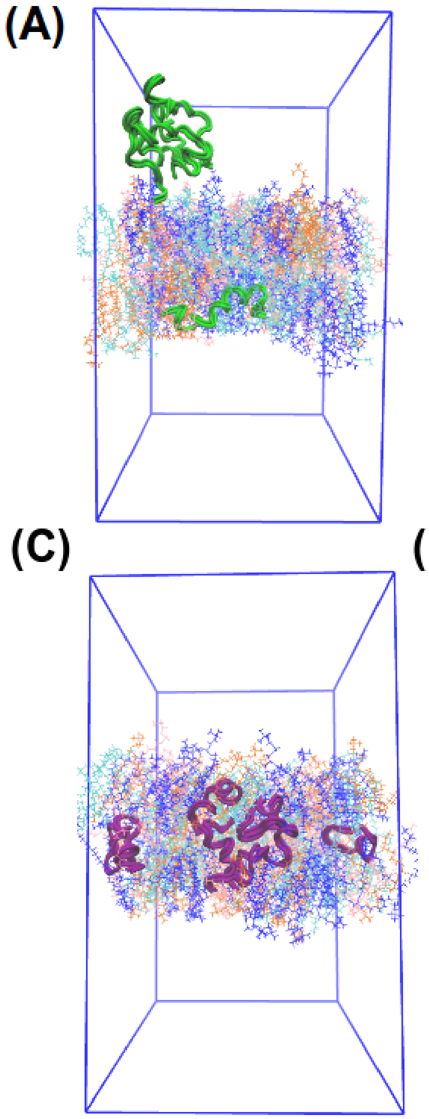

Figure S3: Snapshots of mixed bilayers with one type of peptides: (A) Healthy with Aβ_{1-28} (System 5), (B) Sick with Aβ_{1-28} (System 8),(C) Healthy with Aβ_{26-40} (System 6), (D) Sick with Aβ_{26-40} (System 9, notations of systems is given in Table 2 of the main text). Lipids are represented as lines for bonds. Peptides are shown as ribbons, Aβ_{1-28} is in green, and Aβ_{26-40} is in purple.

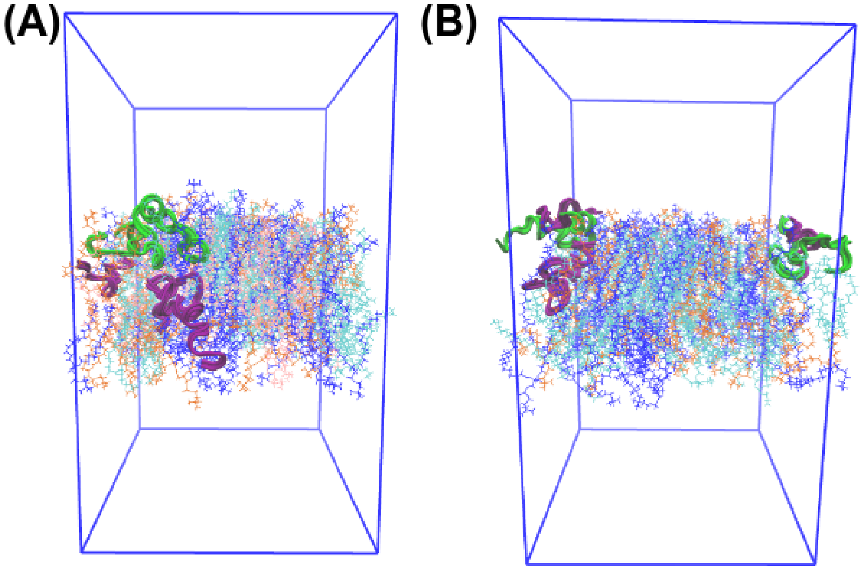

Figure S4: Snapshots of mixed bilayers with two types of peptides: (A) Healthy with $A\beta_{1-28}$ and $A\beta_{26-40}$ (System 7), (B) Sick with $A\beta_{1-28}$ and $A\beta_{26-40}$ (System 10, notations of systems is given in Table 2 of the main text). Lipids are represented as lines for bonds. Peptides are shown as ribbons, $A\beta_{1-28}$ is in green, and $A\beta_{26-40}$ is in purple.

Convergence of the density profiles

In order to ensure convergence of the density profiles, we plot in Figures S5-S16 symmetrized mass density profiles of selected aminoacids during five consecutive 200 ns parts of the total MD trajectory. One can see that in most cases the profiles converge after 400-600 ns, which justify the use of the second half of the microsecond trajectories for the final averaging presented in the main text. Figures S17-S19 show non-symmetrized mass density profiles computed over 500 ns production part of the simulations.

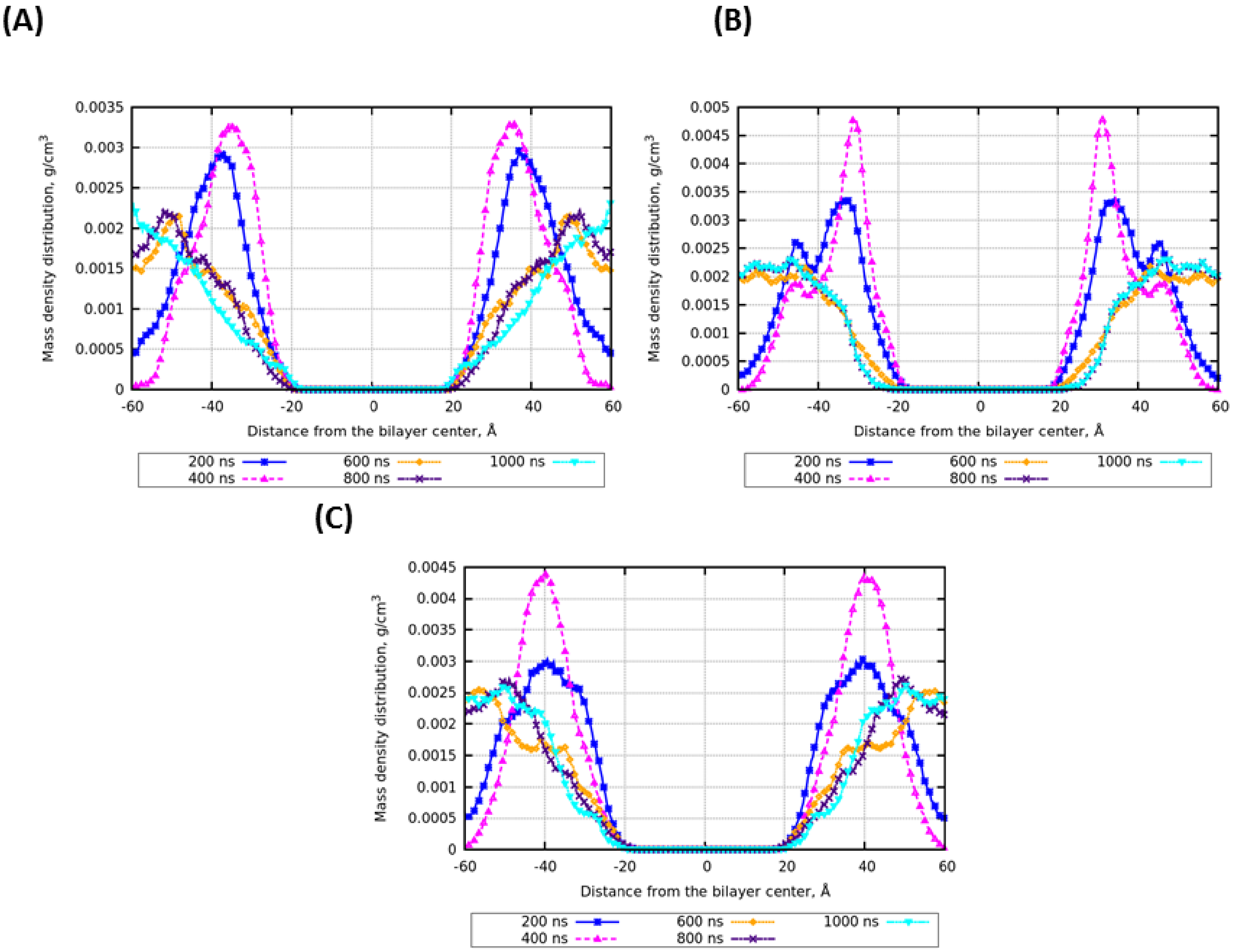

Figure S5. Density profiles of three amino acids for different fragments of the MD trajectory for DMPC - Aβ_{1-28} system (System 1): (A) ASP$_1$. (B) HIS$_{13}$. (C) LYS2$_{28}$

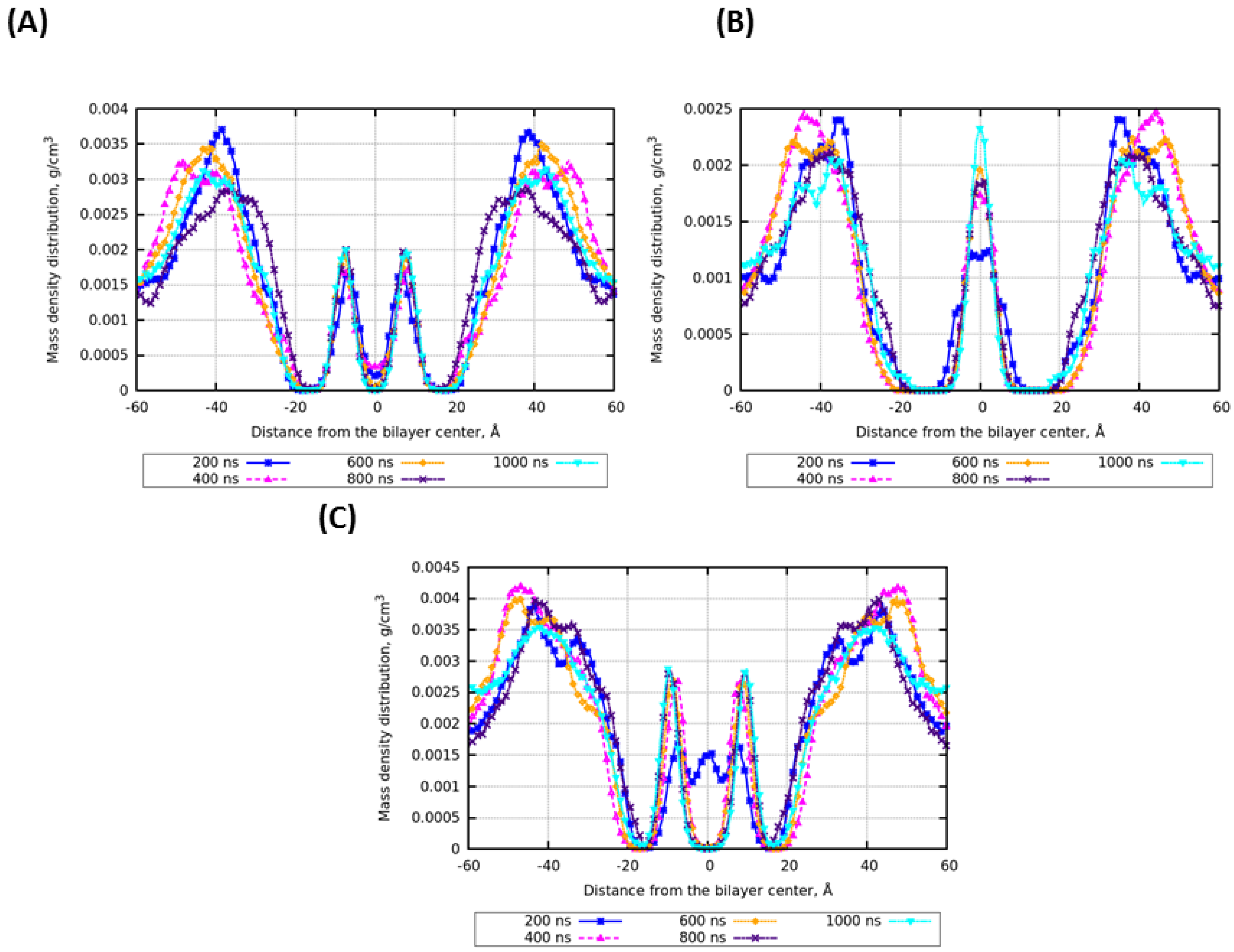

Figure S6. Density profiles of three amino acids for different fragments of the MD trajectory for DMPC - Aβ_{26-40} system (System 2):(A) SER$_2$6. (B) GLY$_{33}$. (C) VAL$_{40}$.

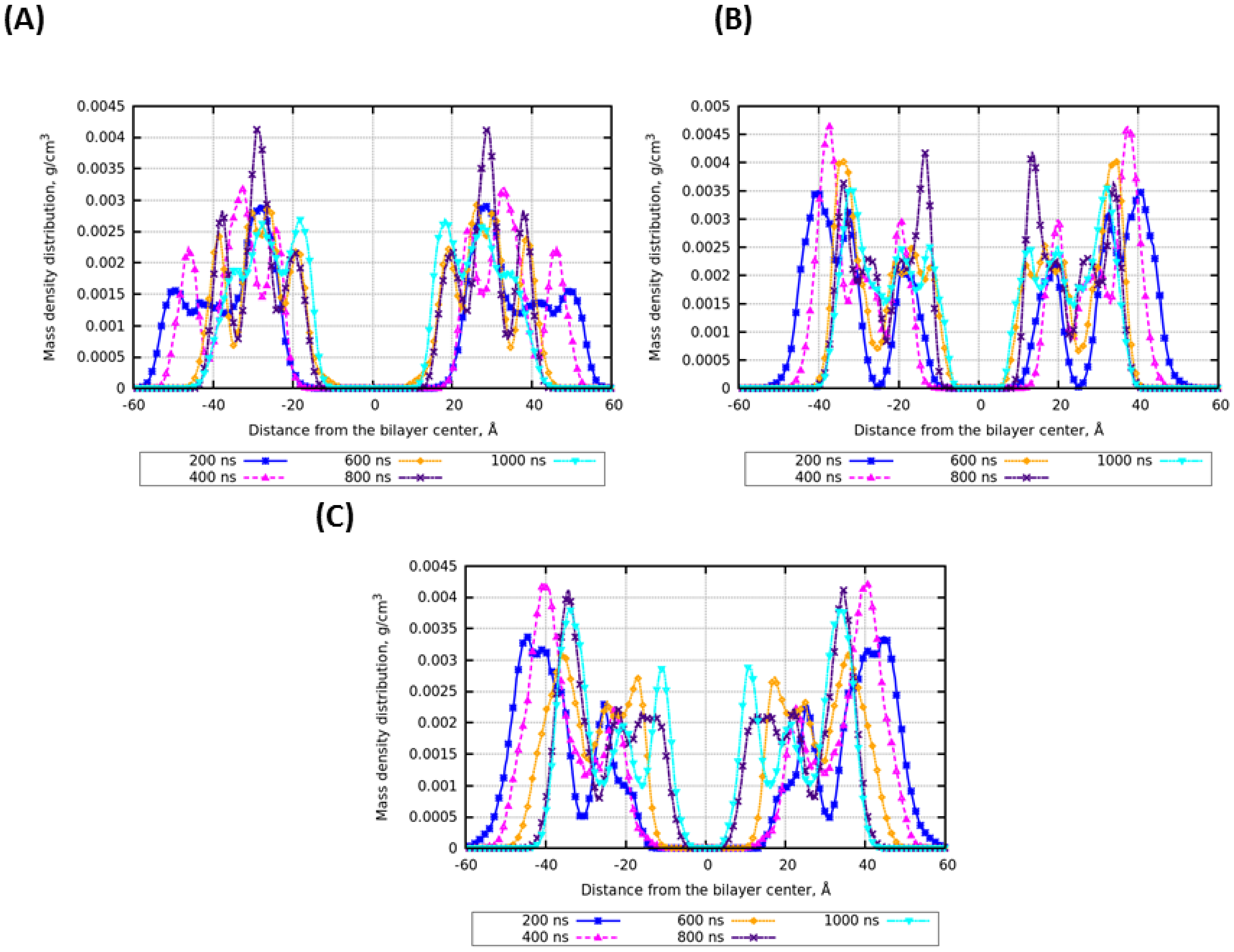

Figure S7. Density profiles of three amino acids for different fragments of the MD trajectory for SDPC - Aβ_{1-28} system (System 3): (A) ASP$_1$. (B) HIS$_{13}$. (C) LYS2$_{28}$.

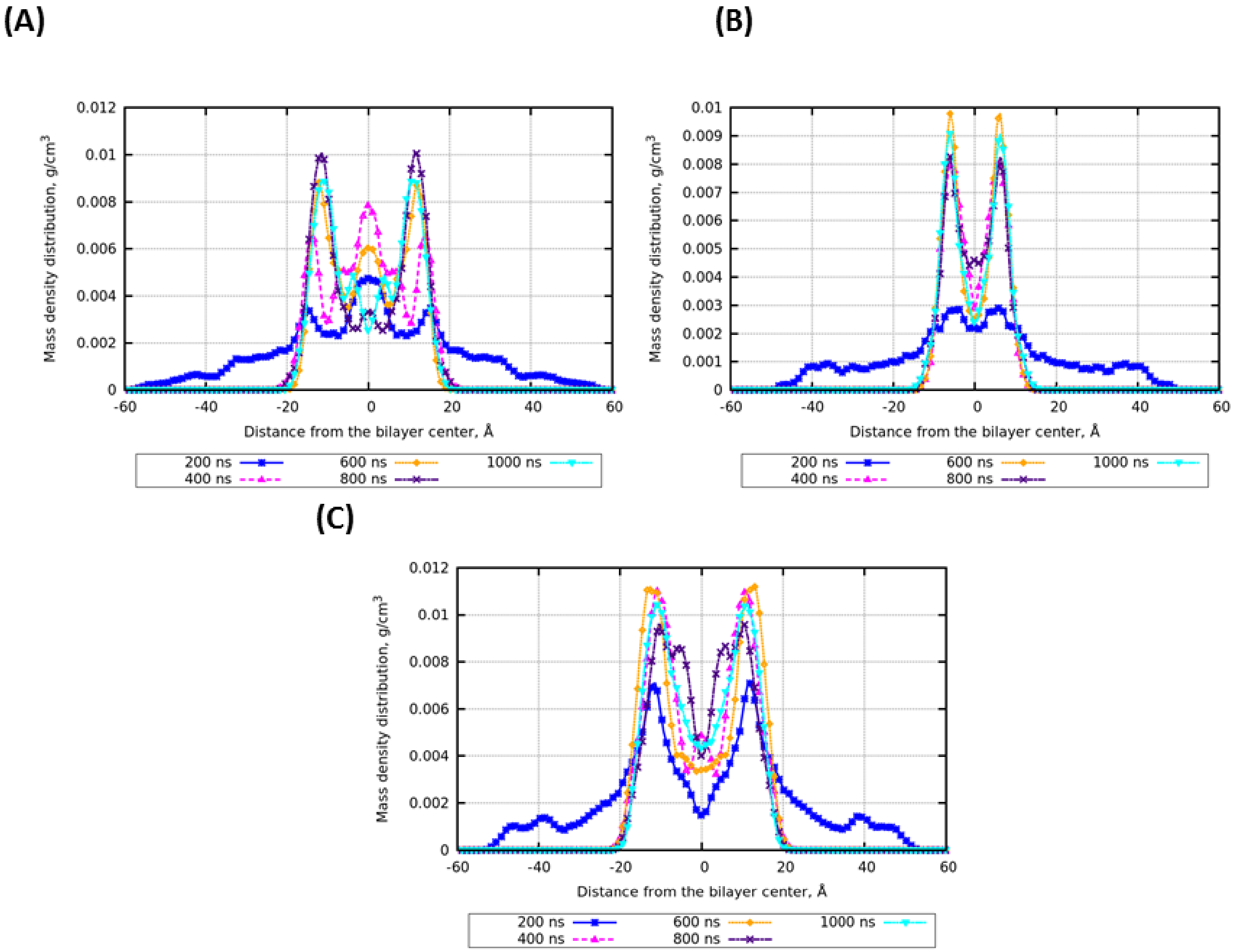

Figure S8. Density profiles of three amino acids for different fragments of the MD trajectory for SDPC - Aβ_{26-40} system (System 4):(A) SER$_2$6. (B) GLY$_{33}$. (C) VAL$_{40}$.

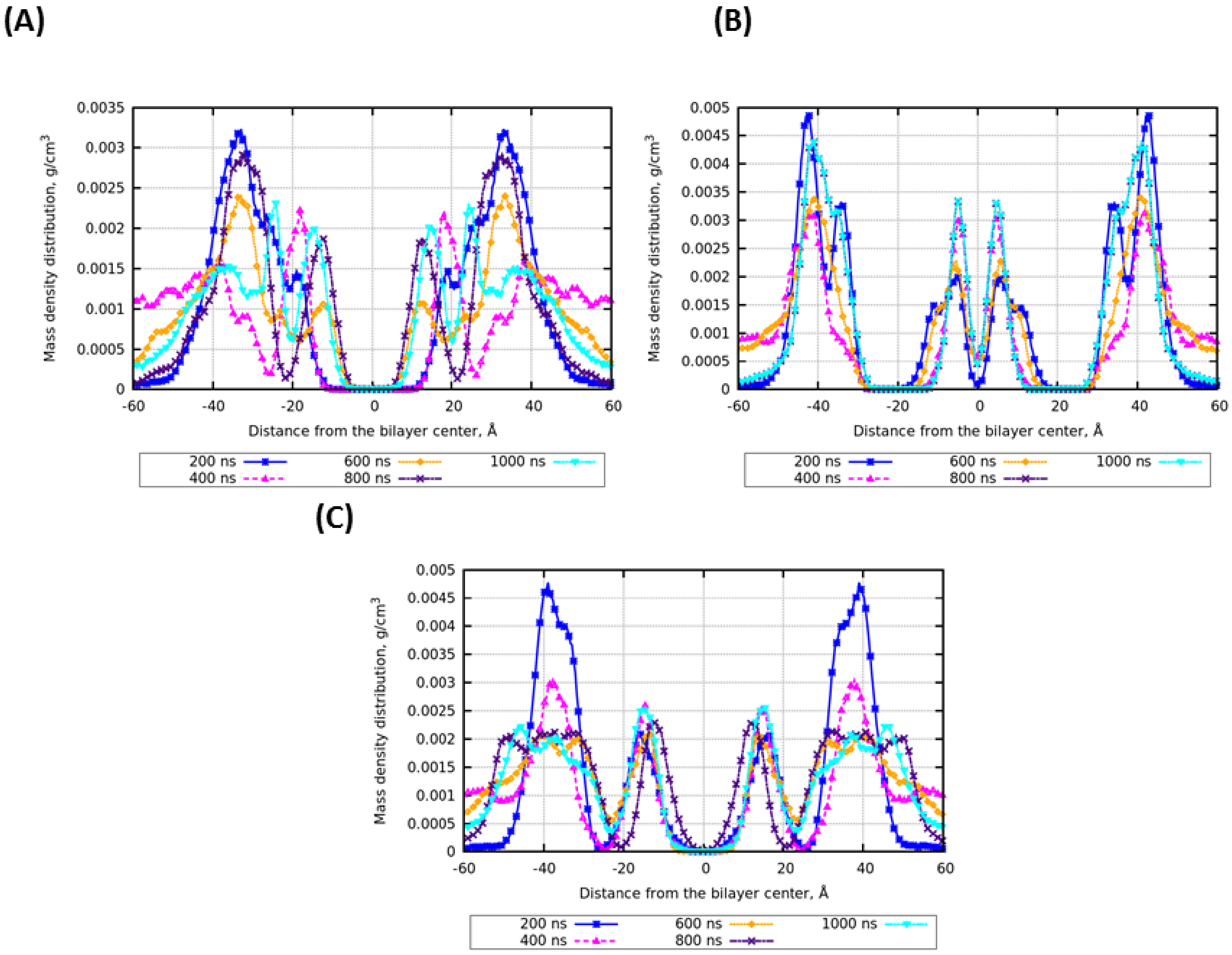

Figure S9. Density profiles of three amino acids for different fragments of the MD trajectory for ”Healthy” bilayer with Aβ_{1-28} (System 5): (A) ASP$_1$. (B) HIS$_{13}$. (C) LYS2$_{28}$.

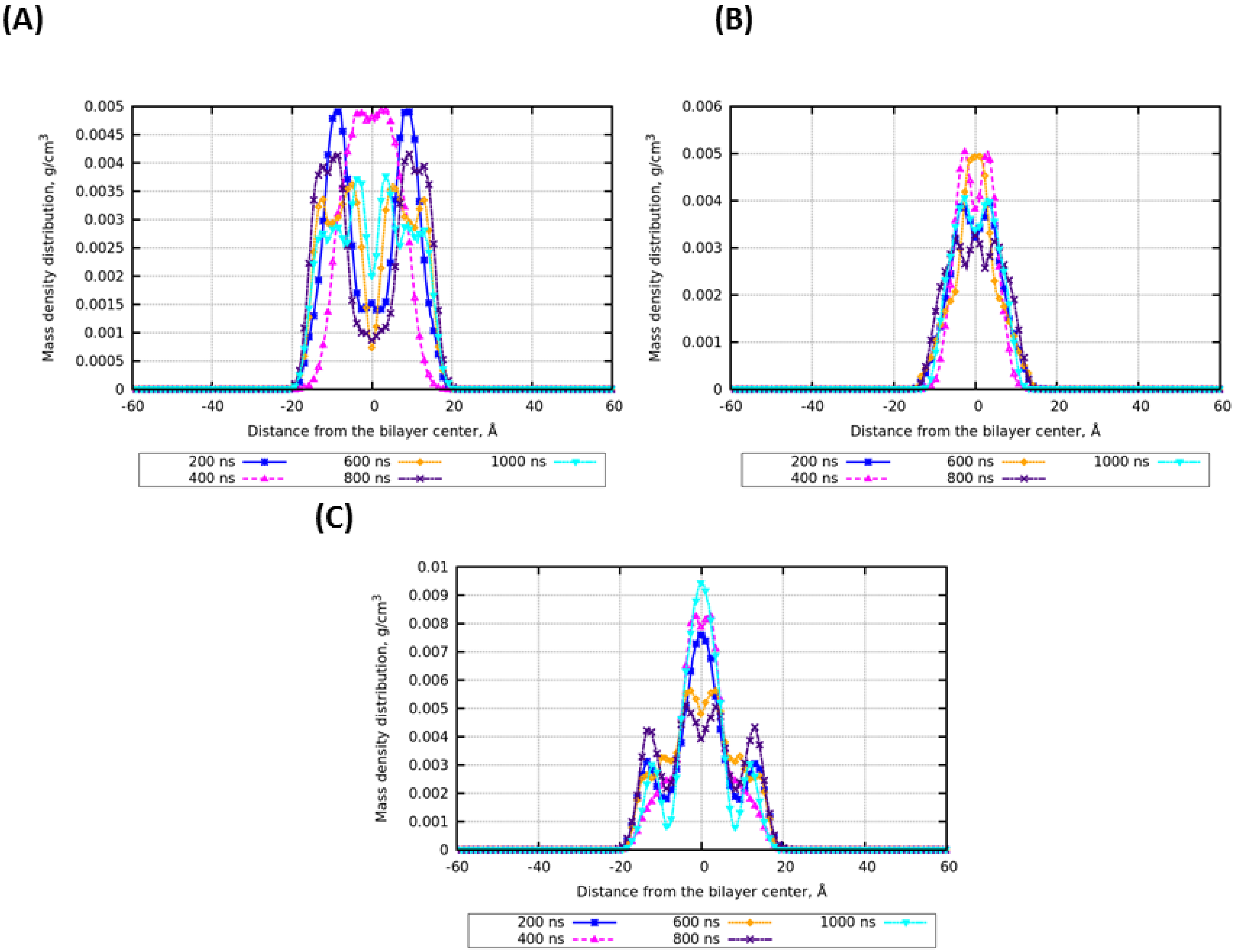

Figure S10. Density profiles of three amino acids for different fragments of the MD trajectory for ”Healthy” bilayer with $A\beta_{26-40}$ (System 6): (A) SER$_2$6. (B) GLY$_{33}$. (C) VAL$_{40}$.

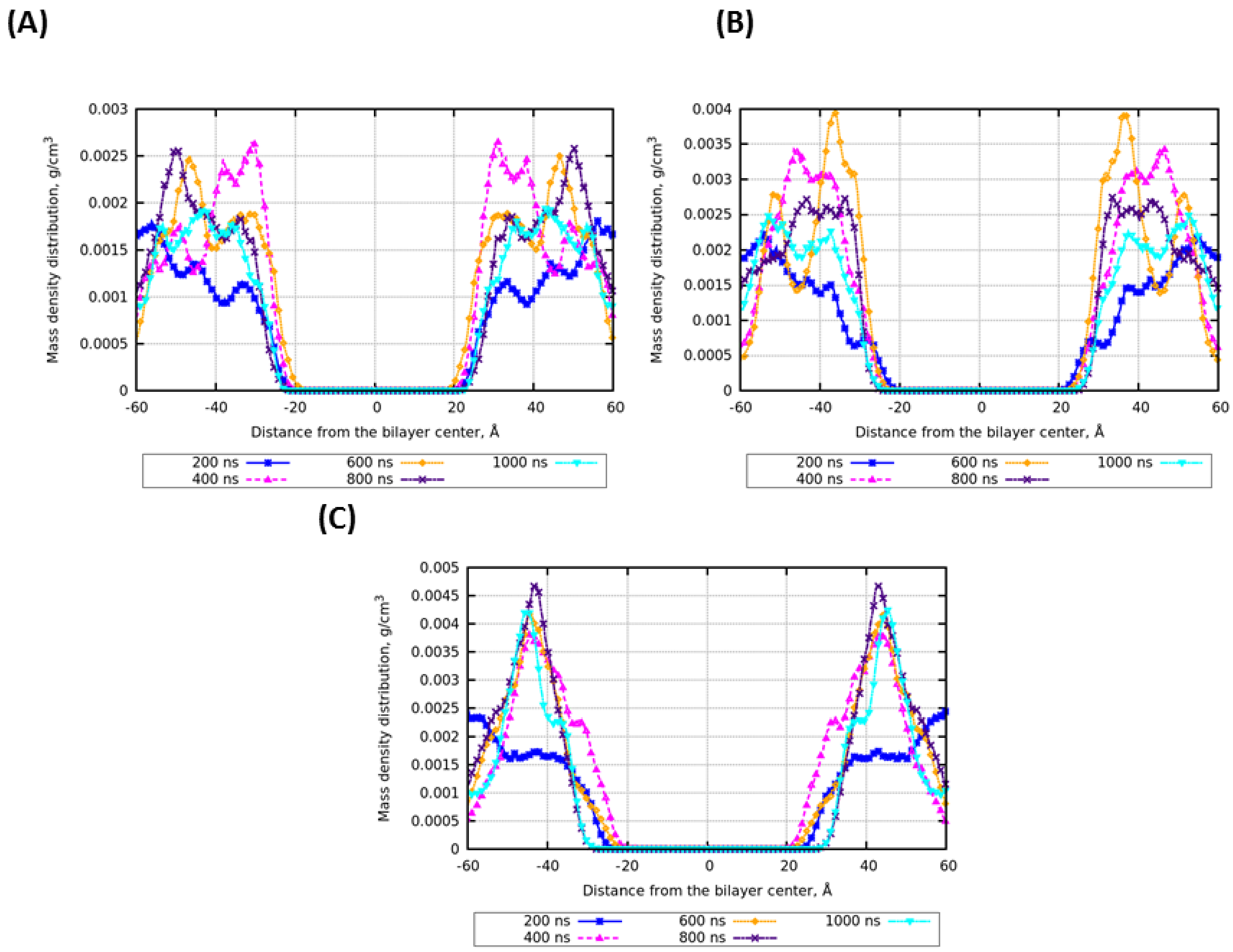

Figure S11. Density profiles of three amino acids for different fragments of the MD trajectory for "Sick" bilayer with Aβ_{1-28} (System 8): (A) ASP_1. (B) HIS_{13}. (C) LYS_{28}.

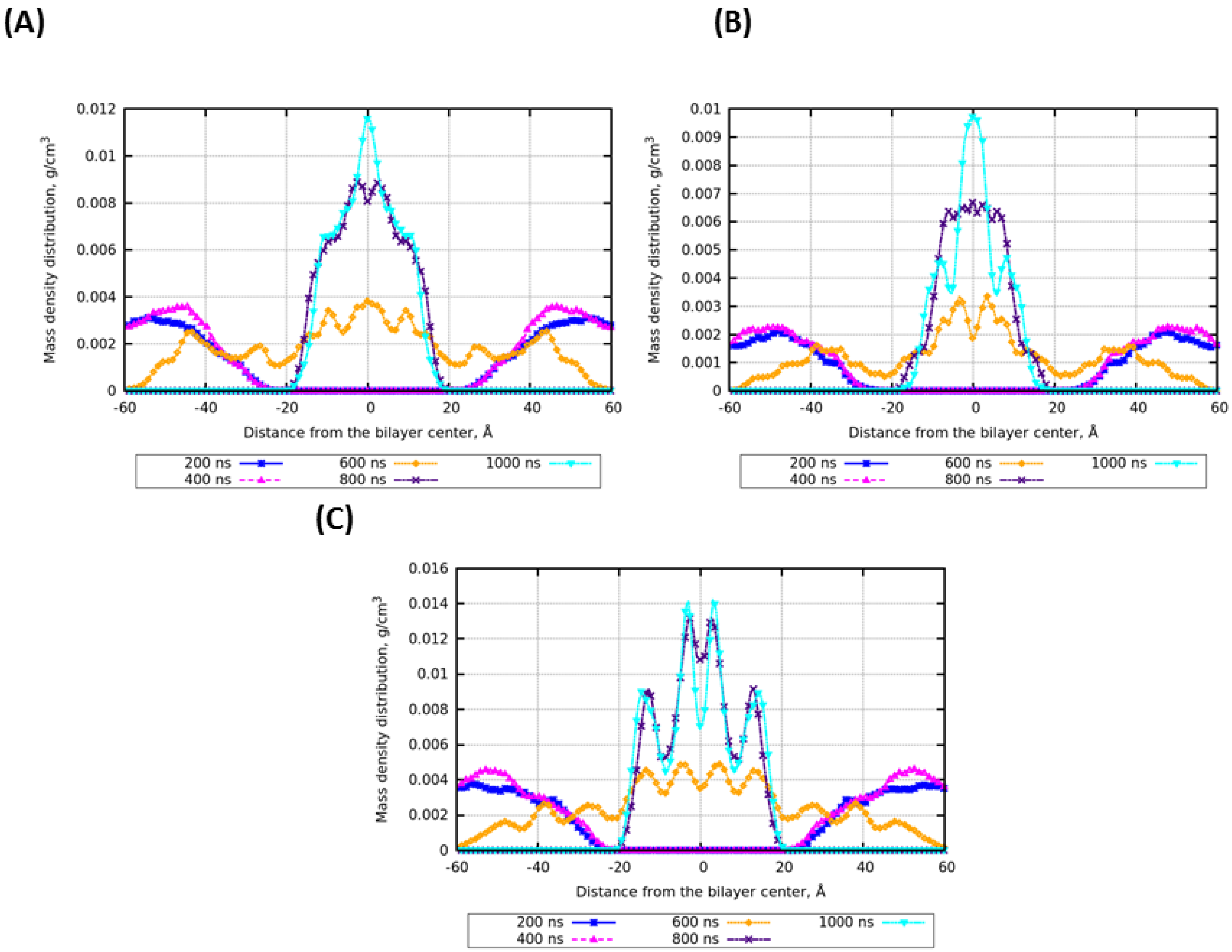

Figure S12: Density profiles of three amino acids for different fragments of the MD trajectory for ”Sick” bilayer with Aβ_{26-40} (System 9): (A) SER$_2$6. (B) GLY$_{33}$. (C) VAL$_{40}$.

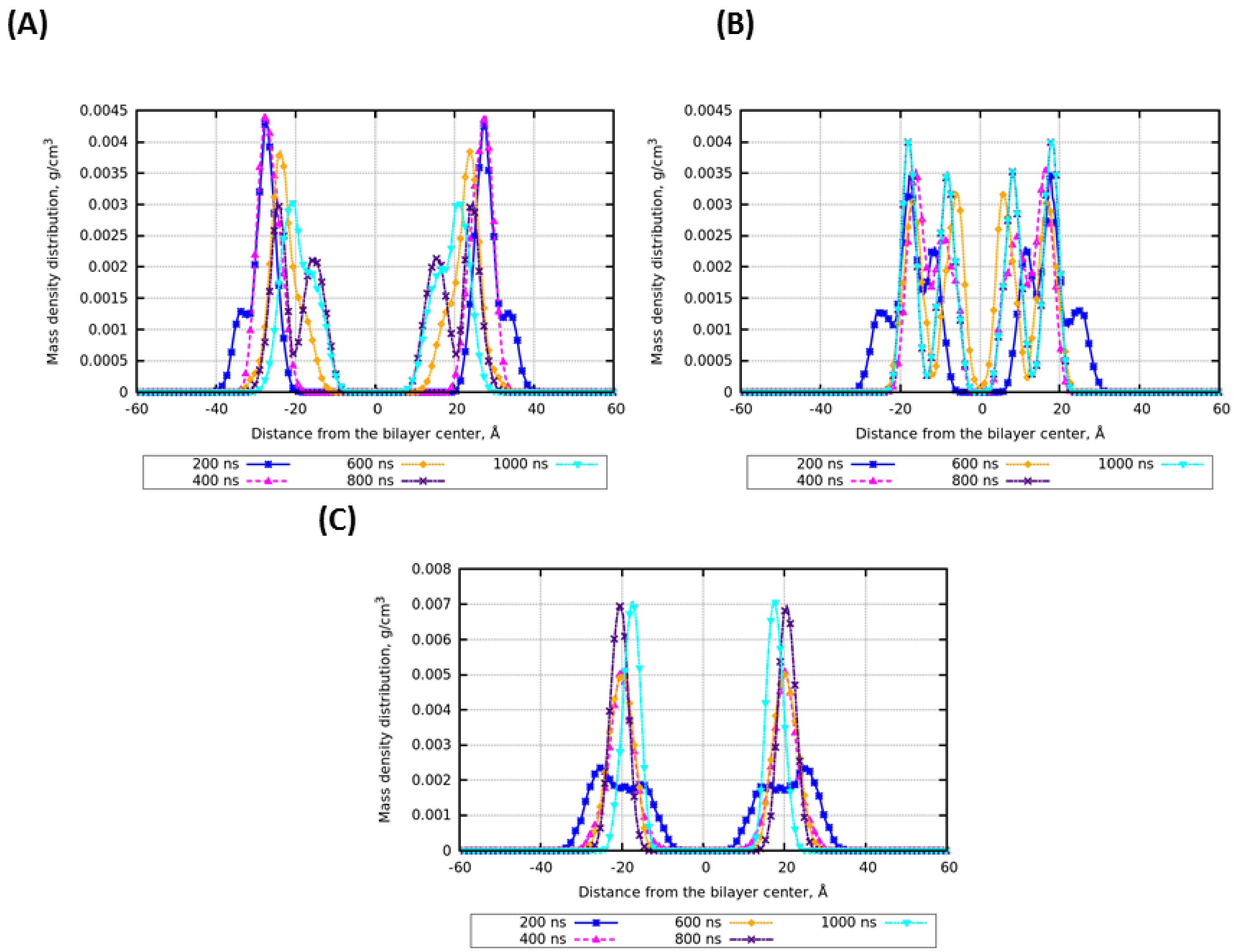

Figure S13. Density profiles of three amino acids of Aβ_{1-28} peptide for different fragments of the MD trajectory for "Healthy" bilayer with both types of peptides (System 7): (A) ASP_1. (B) HIS_{13}. (C) $LYS2_{26}$.

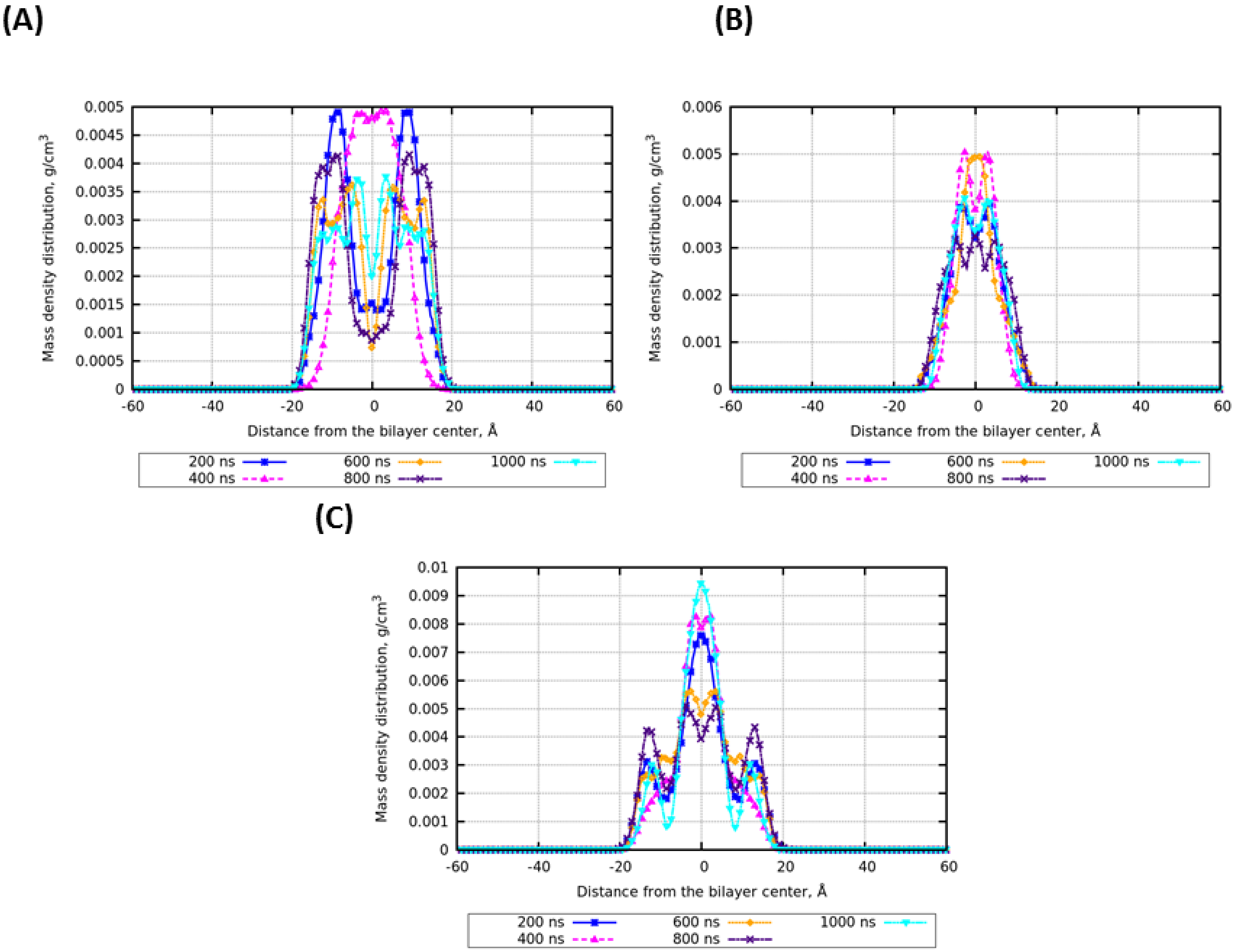

Figure S14. Density profiles of three amino acids of Aβ_{26-40} peptide for different fragments of the MD trajectory for "Healthy" bilayer with both types of peptides (System 7):(A) SER$_2$6. (B) GLY$_{33}$. (C) VAL$_{40}$.

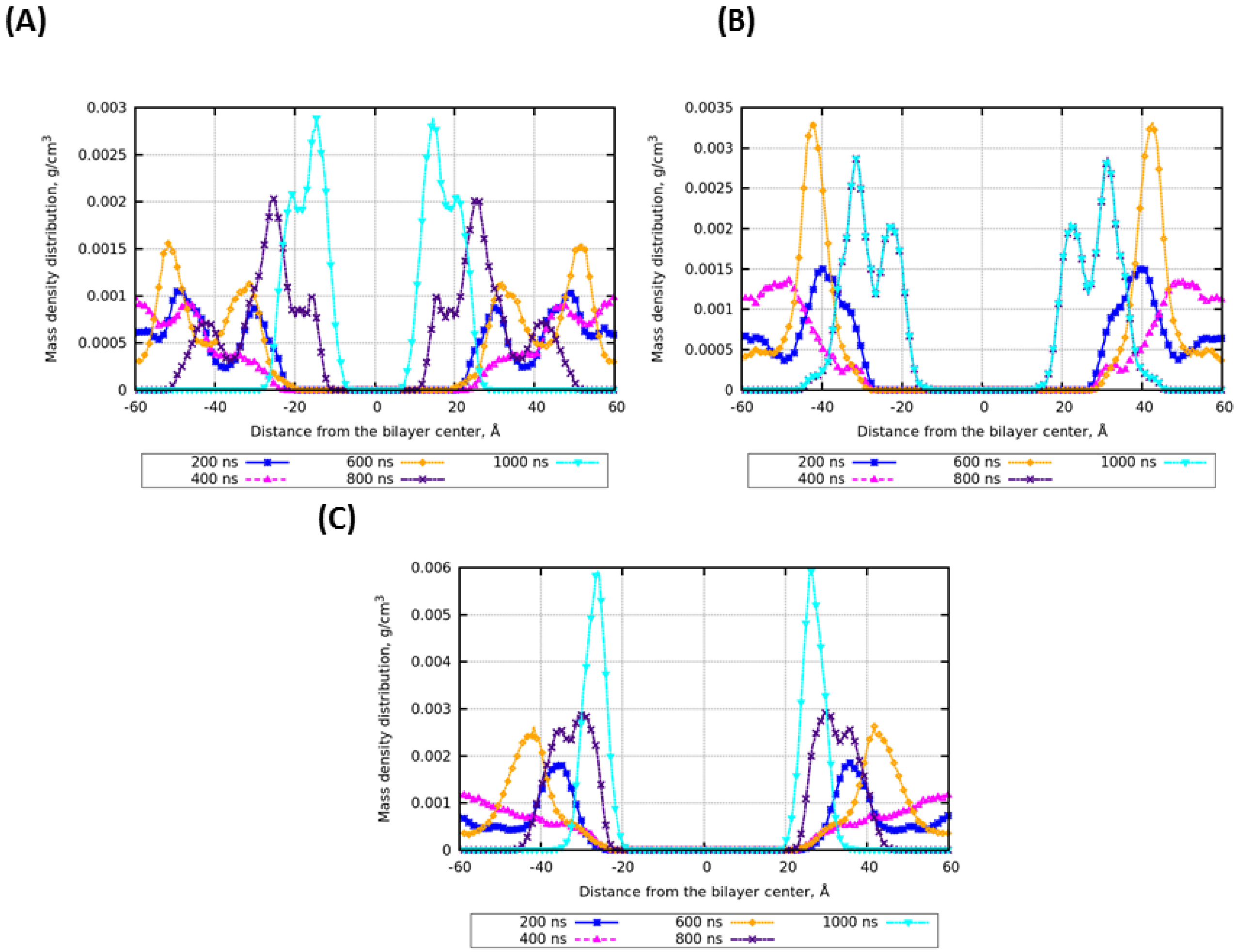

Figure S15. Density profiles of three amino acids of Aβ_{1-28} peptide for different fragments of the MD trajectory for "Sick" bilayer with both types of peptides (System 10): (A) ASP_1. (B) HIS_{13}. (C) $LYS2_{26}$.

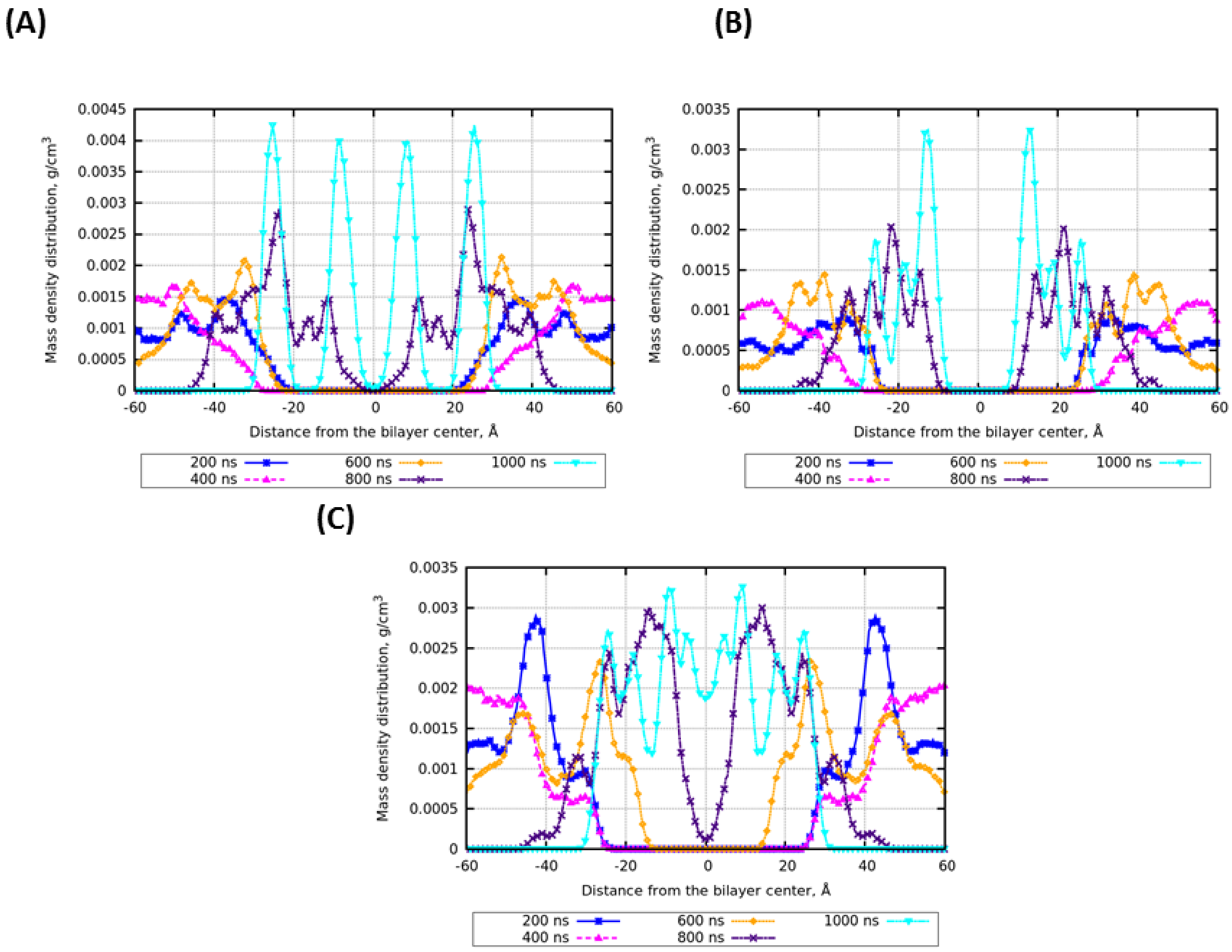

Figure S16. Density distribution profiles of three amino acids of Aβ_{26-40} peptide for different fragments of the MD trajectory for "Sick" bilayer with both types of peptides (System 10):(A) $SER_2$6. (B) GLY_{33}. (C) VAL_{40}.

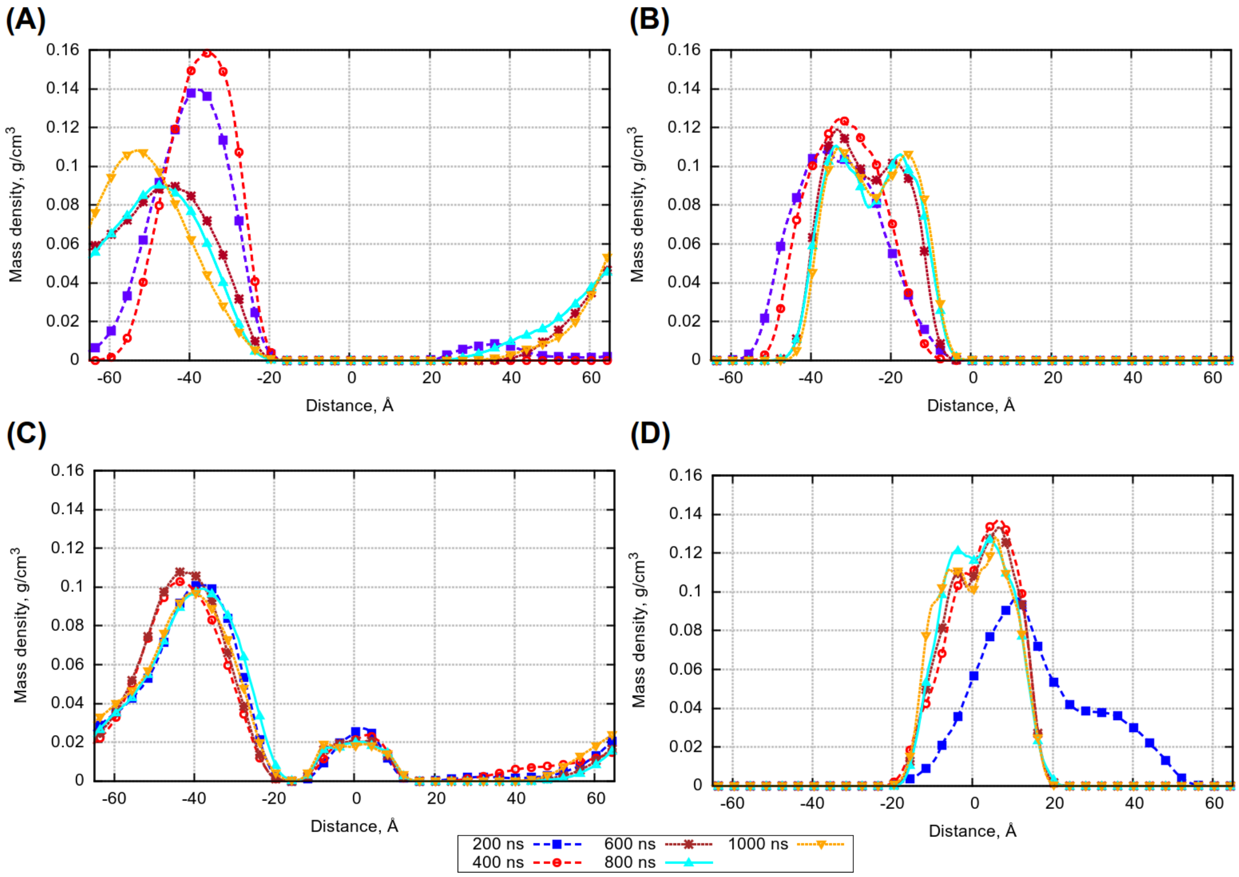

Figure S17. Non-symmetrized mass density profiles for peptides. (A) DMPC and A$\beta(1-28)$ (B) SDPC and A$\beta(1-28)$ (C) DMPC and A$\beta(26-40)$ (D) SDPC and A$\beta(26-40)$

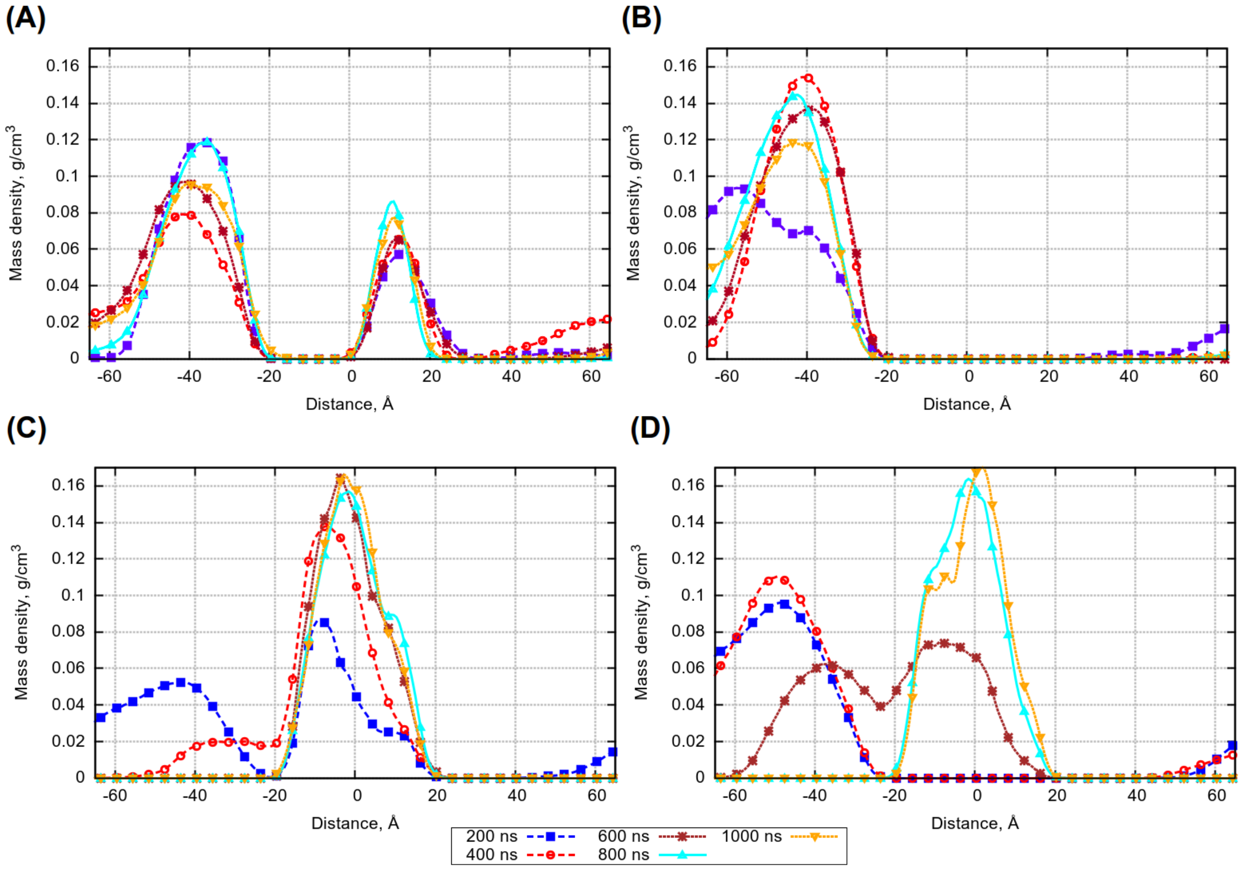

Figure S18. Non-symmetrized mass density profiles for mixed systems containing one kind of peptides (contributions from peptides). (A) Healthy and A$\beta(1-28)$ (B) Sick and A$\beta(1-28)$ (C) Healthy and A$\beta(26-40)$ (D) Sick and A$\beta(26-40)$

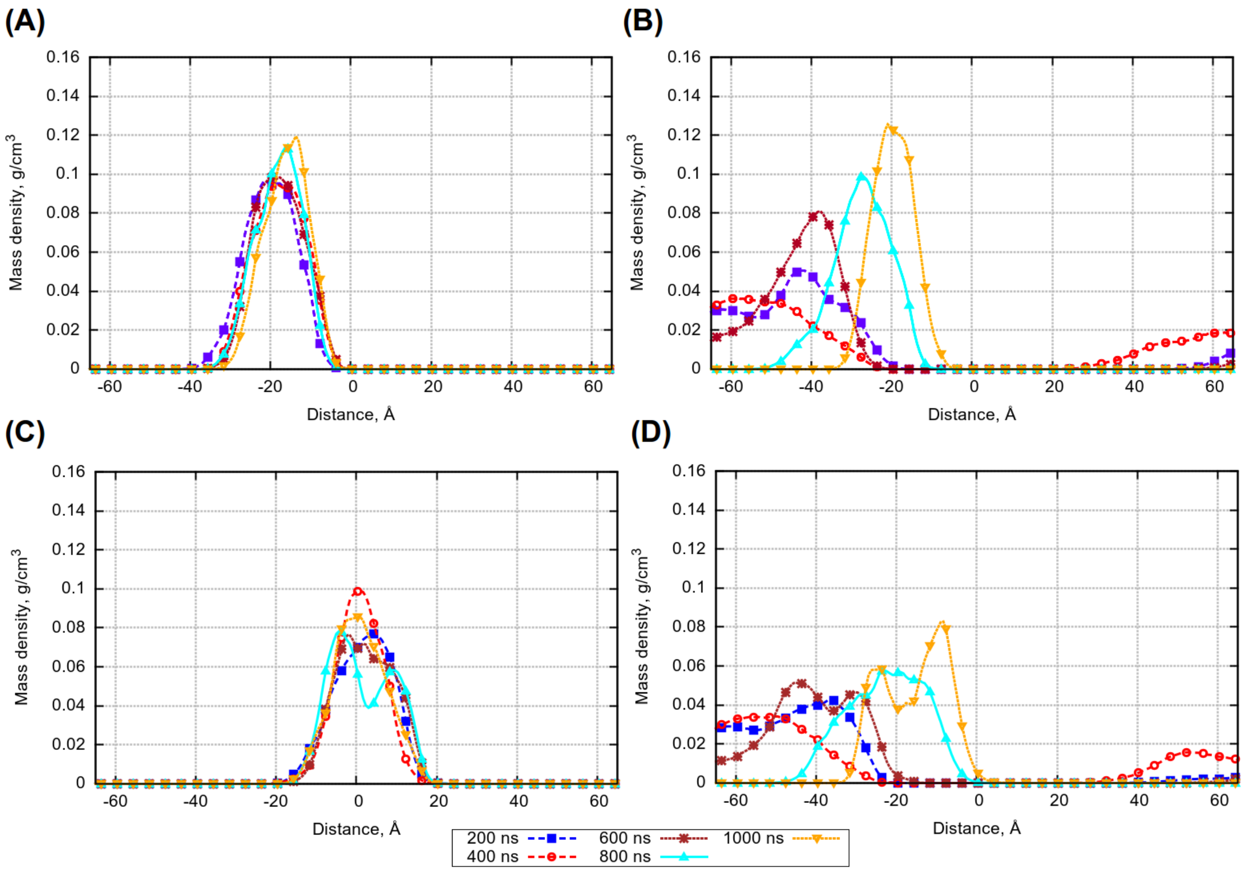

Figure S19. Non-symmetrized mass density profiles for mixed systems containing both peptides (contributions from peptides). (A) Healthy and A$\beta(1-28)$ (B) Sick and A$\beta(1-28)$ (C) Healthy and A$\beta(26-40)$ (D) Sick and A$\beta(26-40)$

Contact maps through the whole simulation time

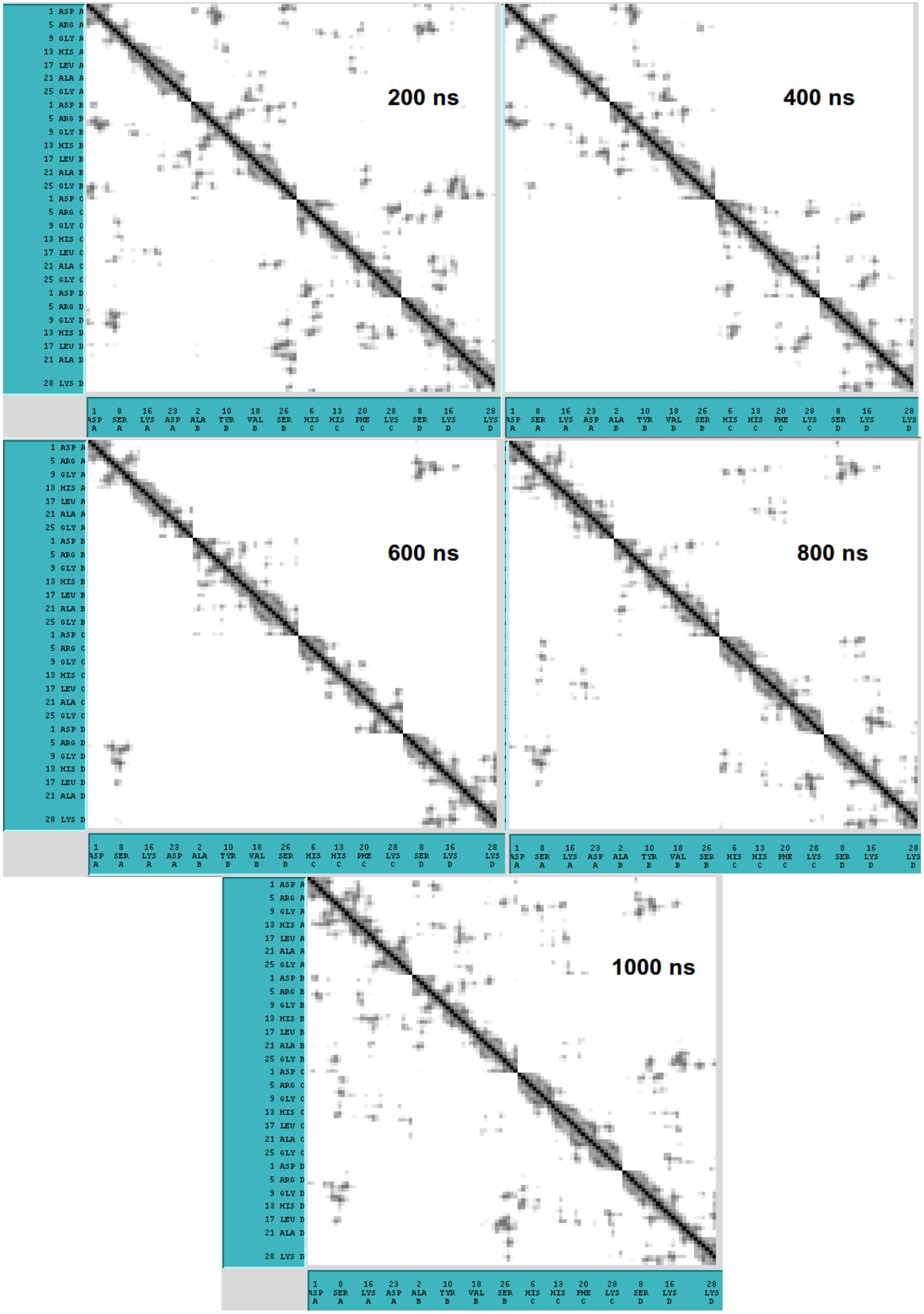

Figure S20. Contact maps computed during five 200 ns time intervals for the system containing 14:0-14:0 PC lipids and Aβ_{1-28}. Letters A-D given on x and y represent different peptides.

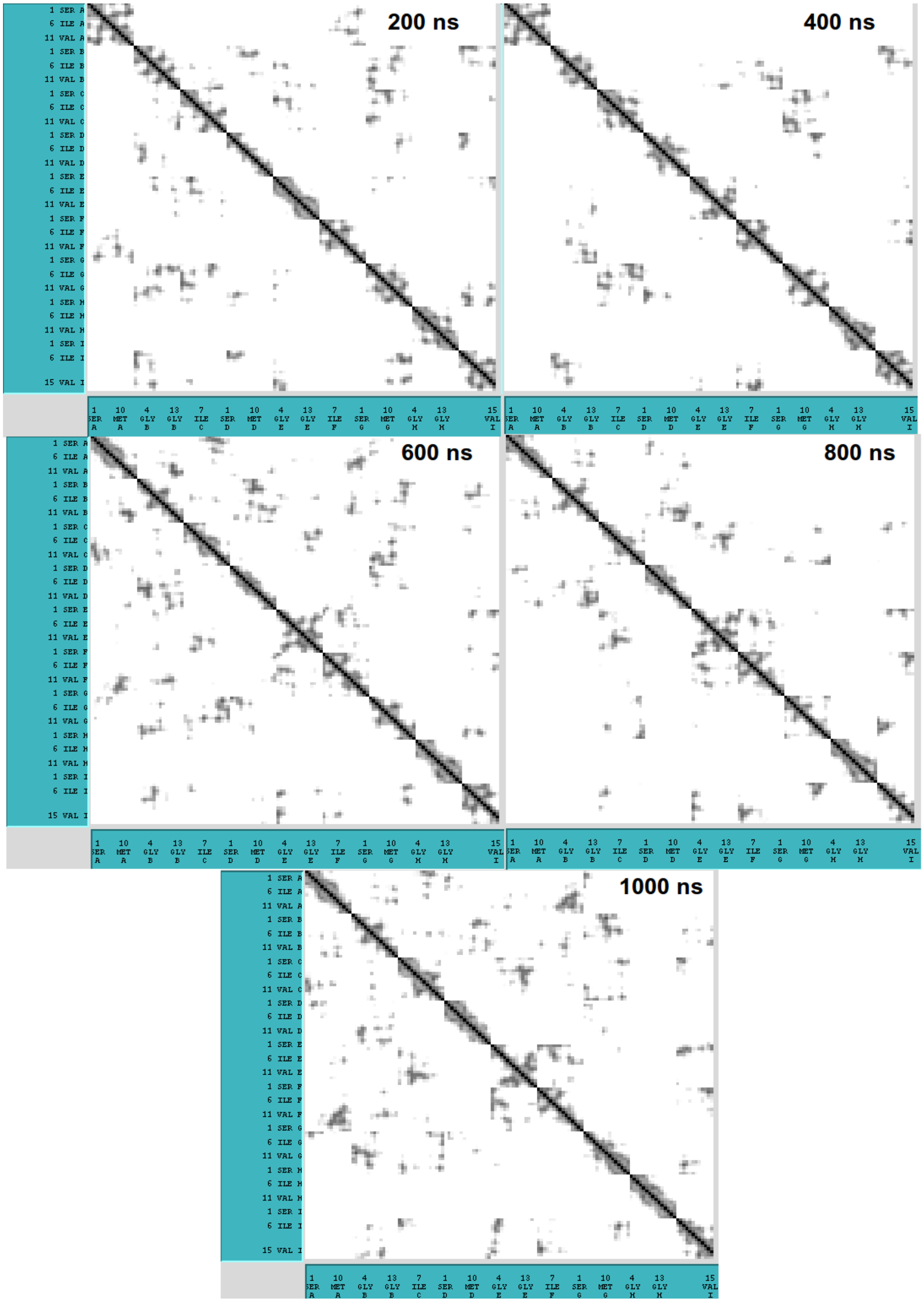

Figure S21. Contact maps computed during five 200 ns time intervals for the system containing 14:0 - 14:0 PC lipids and Aβ_{26-40}. Letters A-I given on x and y axes represent different peptides.

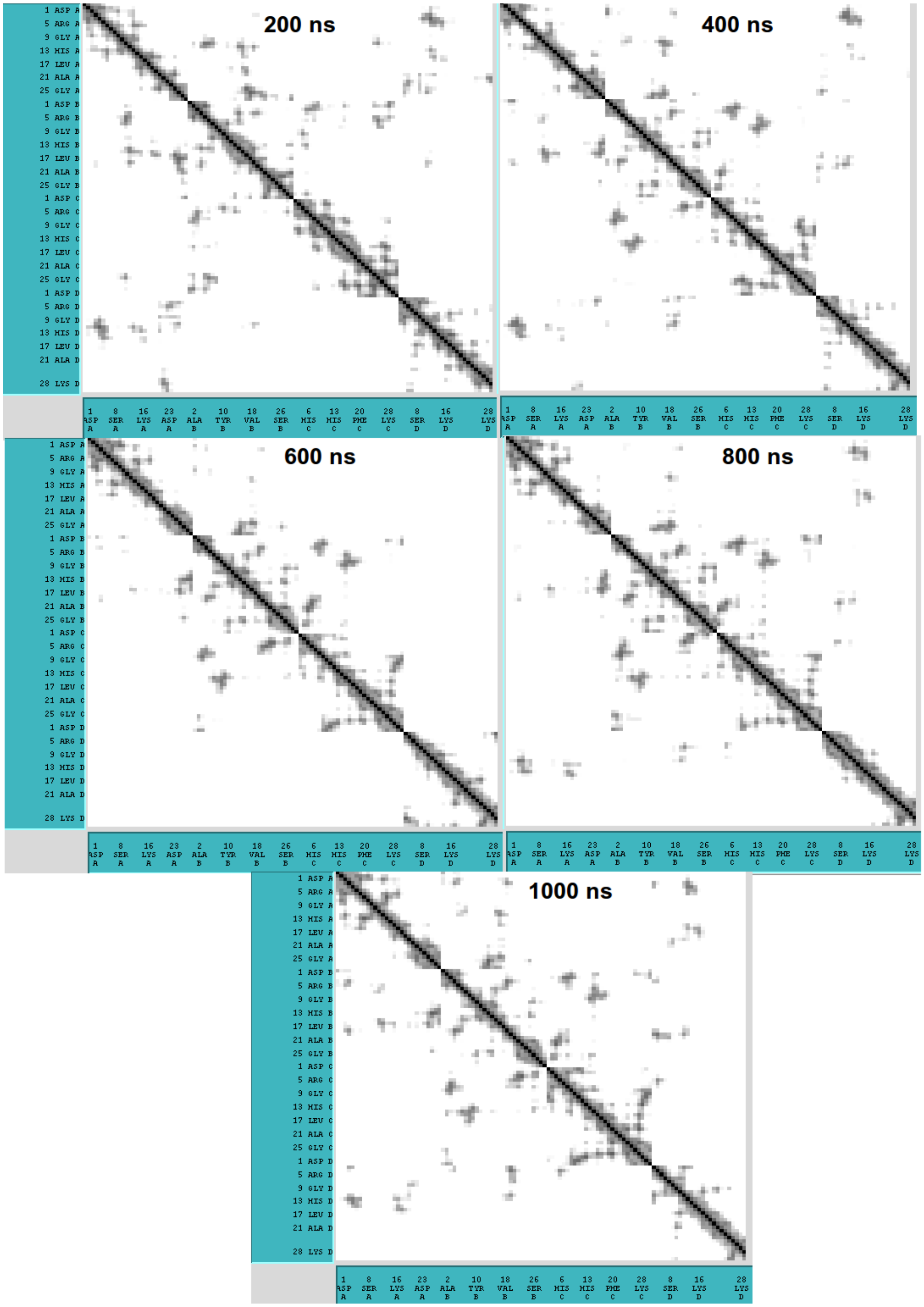

Figure S22. Contact maps computed during five 200 ns time intervals for the system containing 18:0 - 22:6 PC lipids and Aβ_{1-28}. Letters A-D given on x and y axes represent different peptides.

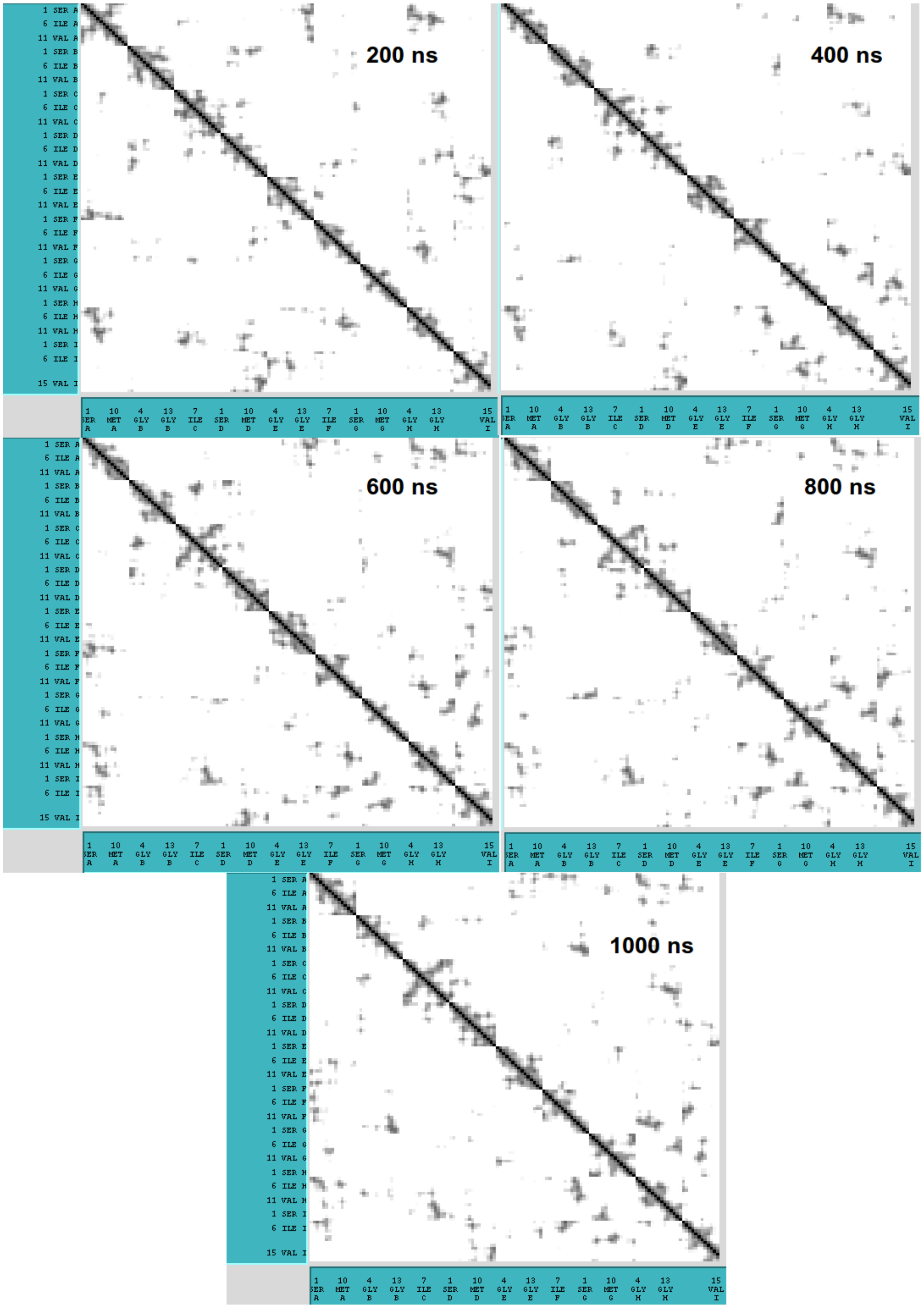

Figure S23. Contact maps computed during five 200 ns time intervals for the system containing 18:0 - 22:6 PC lipids and Aβ_{26-40}. Letters A-D given on x and y axes represent different peptides.

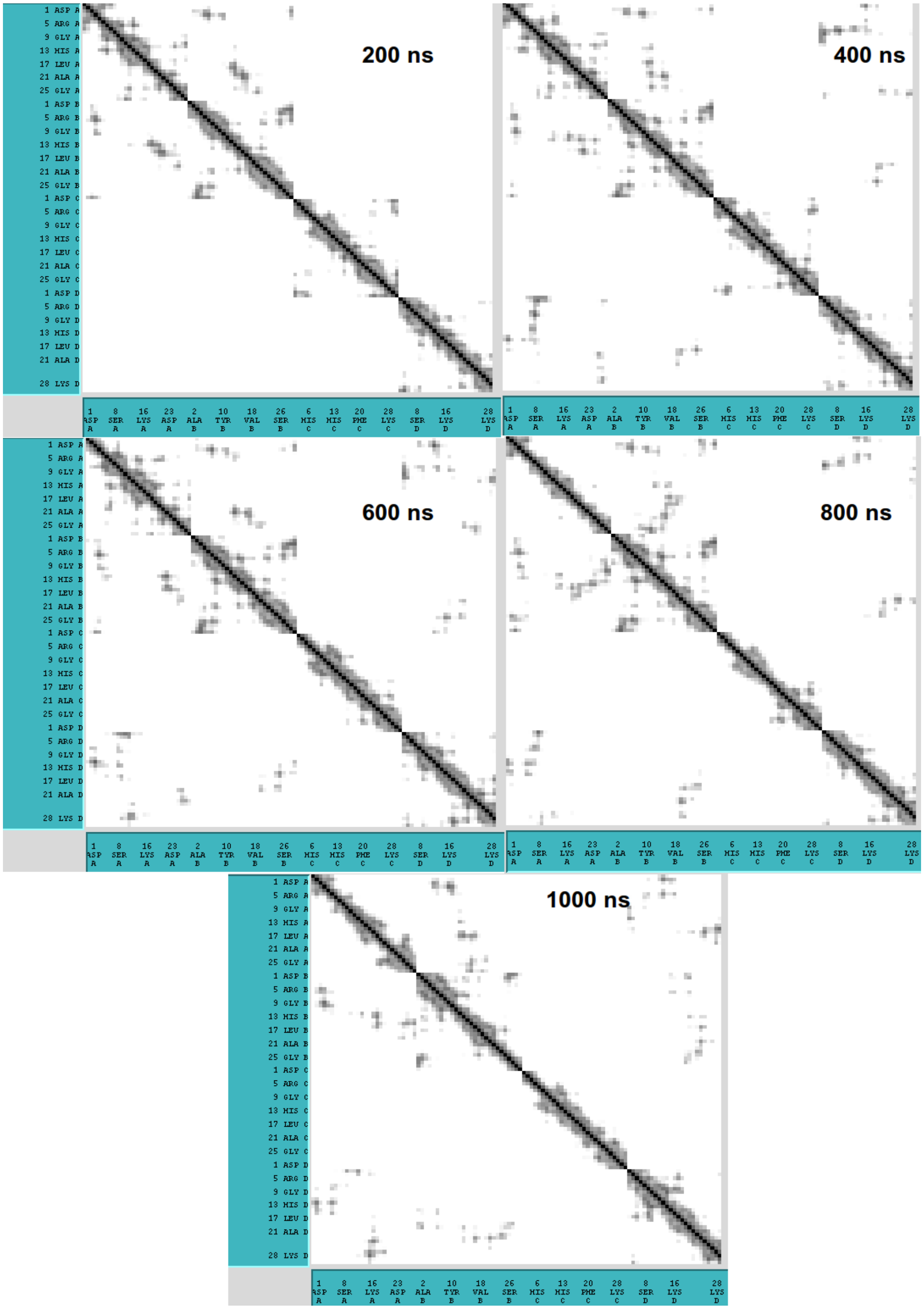

Figure S24. Contact maps computed during five 200 ns time intervals for mixed "normal" bilayer containing Aβ_{1-28}. Letters A-D given on x and y axes represent different peptides.

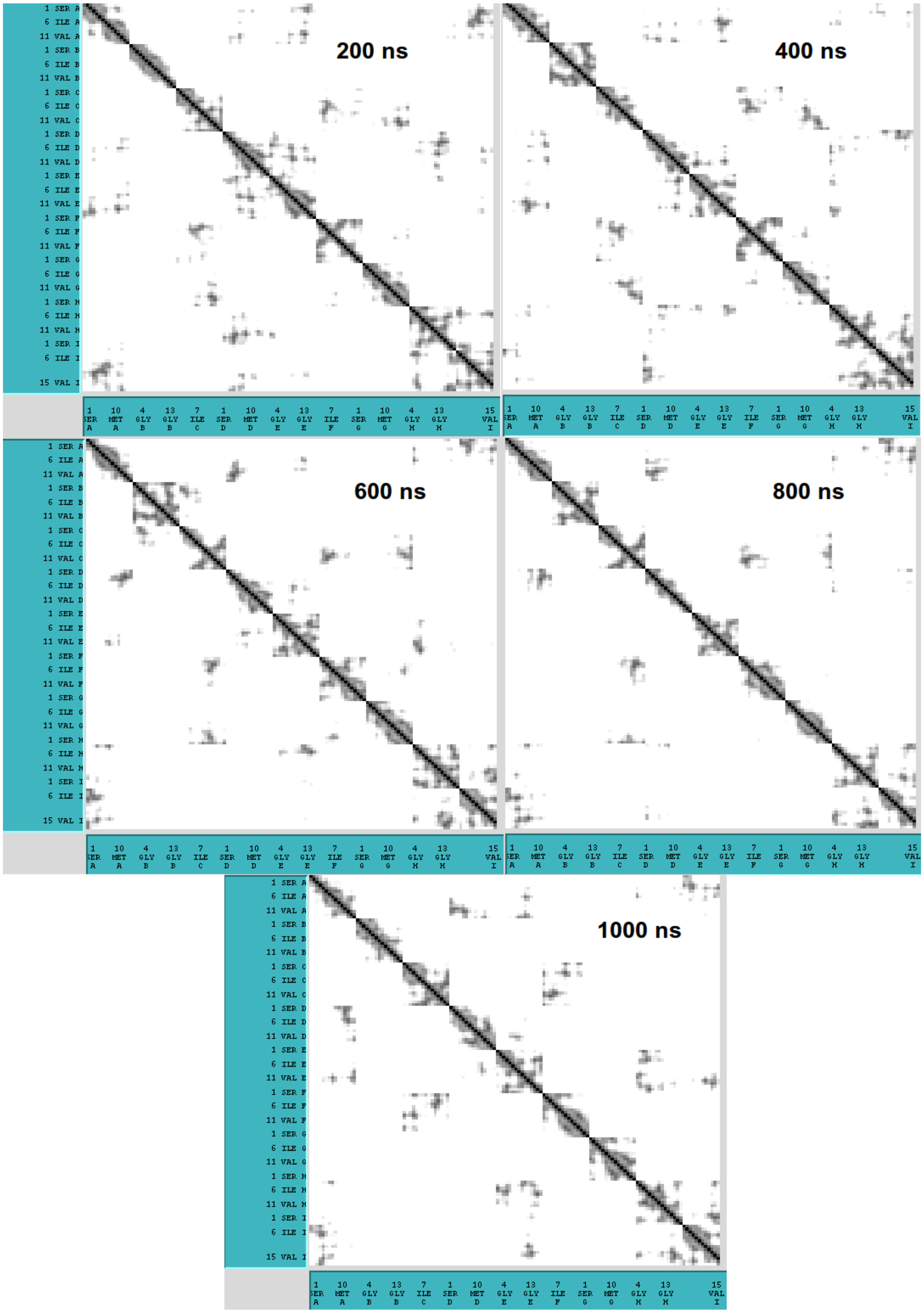

Figure S25. Contact maps computed during five 200 ns time intervals for mixed "normal" membrane containing Aβ_{26-40}. Letters A-I listed on x and y axis represent different peptides.

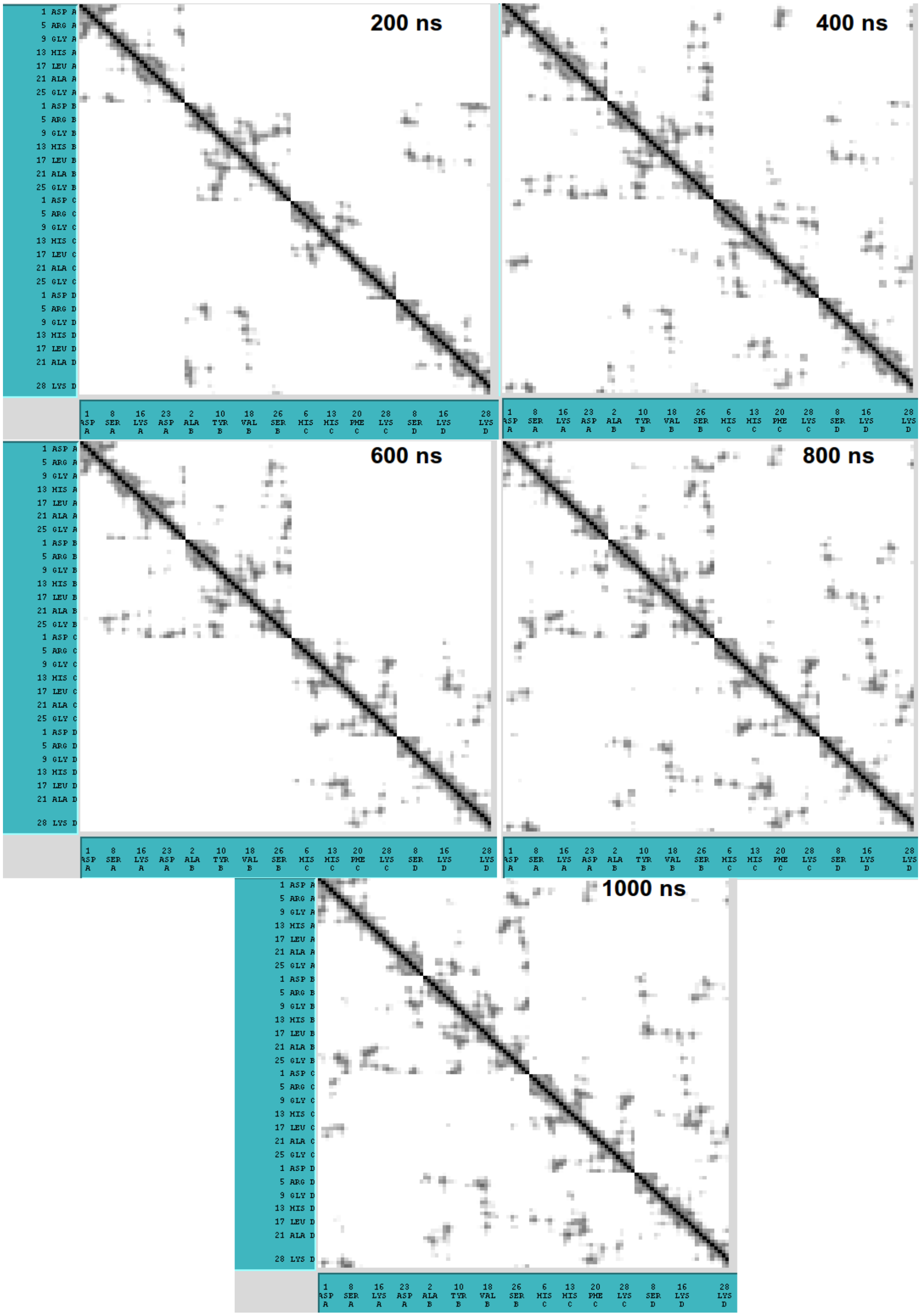

Figure S26. Contact maps computed during five 200 ns time intervals for mixed "AD" membrane containing Aβ_{1-28}. Letters A-D given on x and y axis represent different peptides.

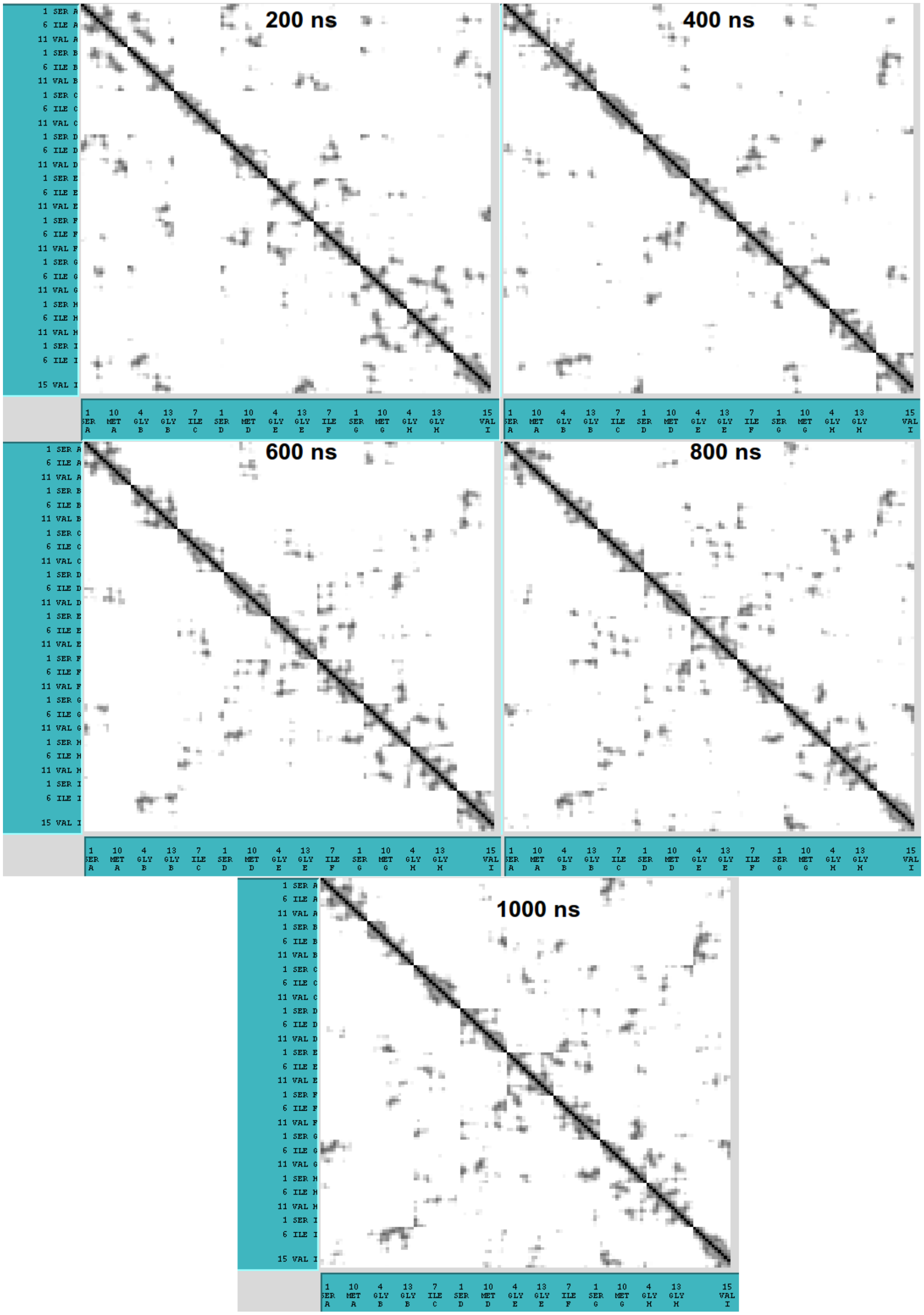

Figure S27. Contact maps computed during five 200 ns time intervals for mixed "AD" membrane containing Aβ_{26-40}. Letters A-I given on x and y axis represent different peptides.

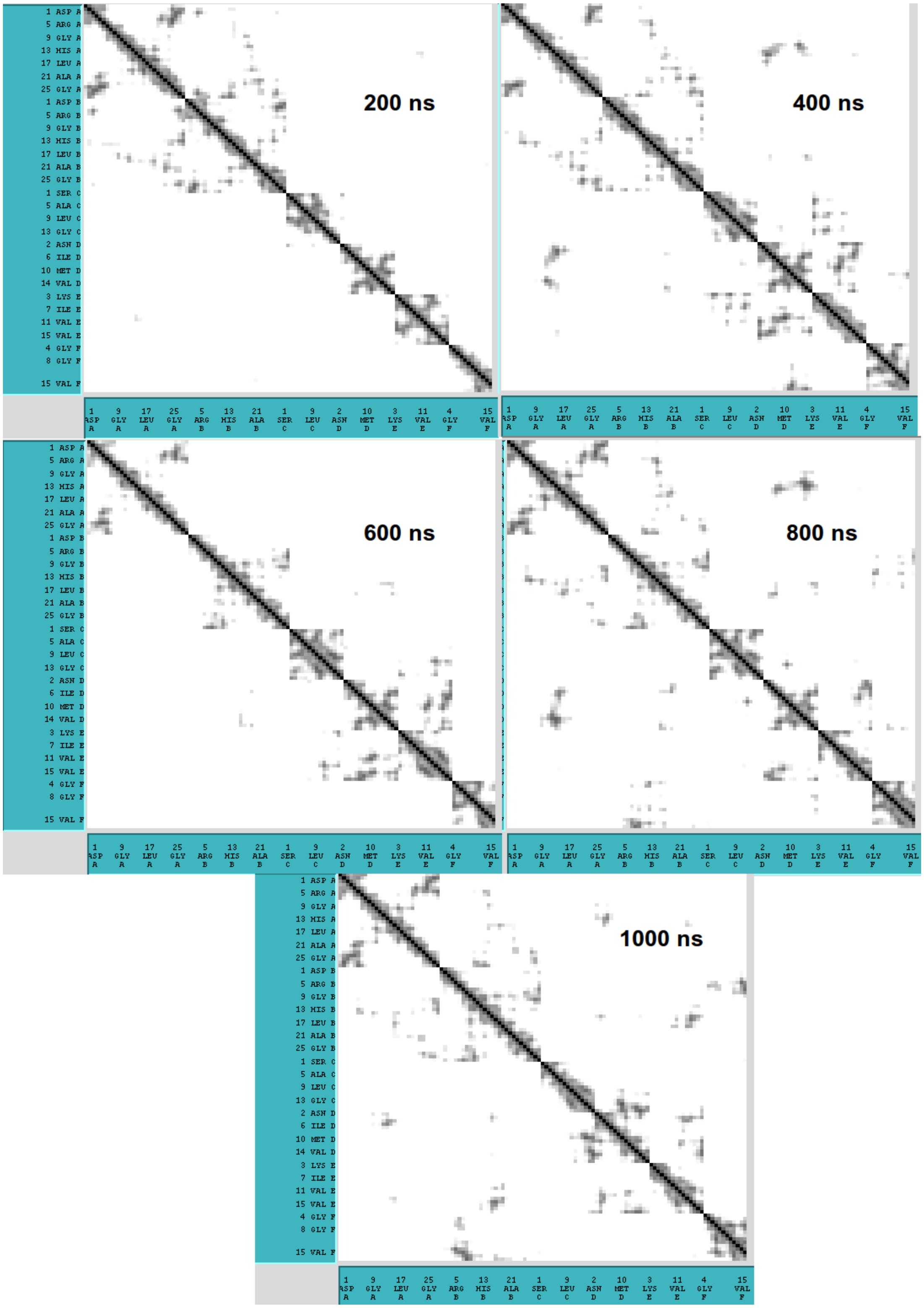

Figure S28. Contact maps computed during five 200 ns time intervals for mixed "normal" membrane containing both $A\beta_{1-28}$ and $A\beta_{26-40}$. Letters A-F given on x and y axis represent different peptides.

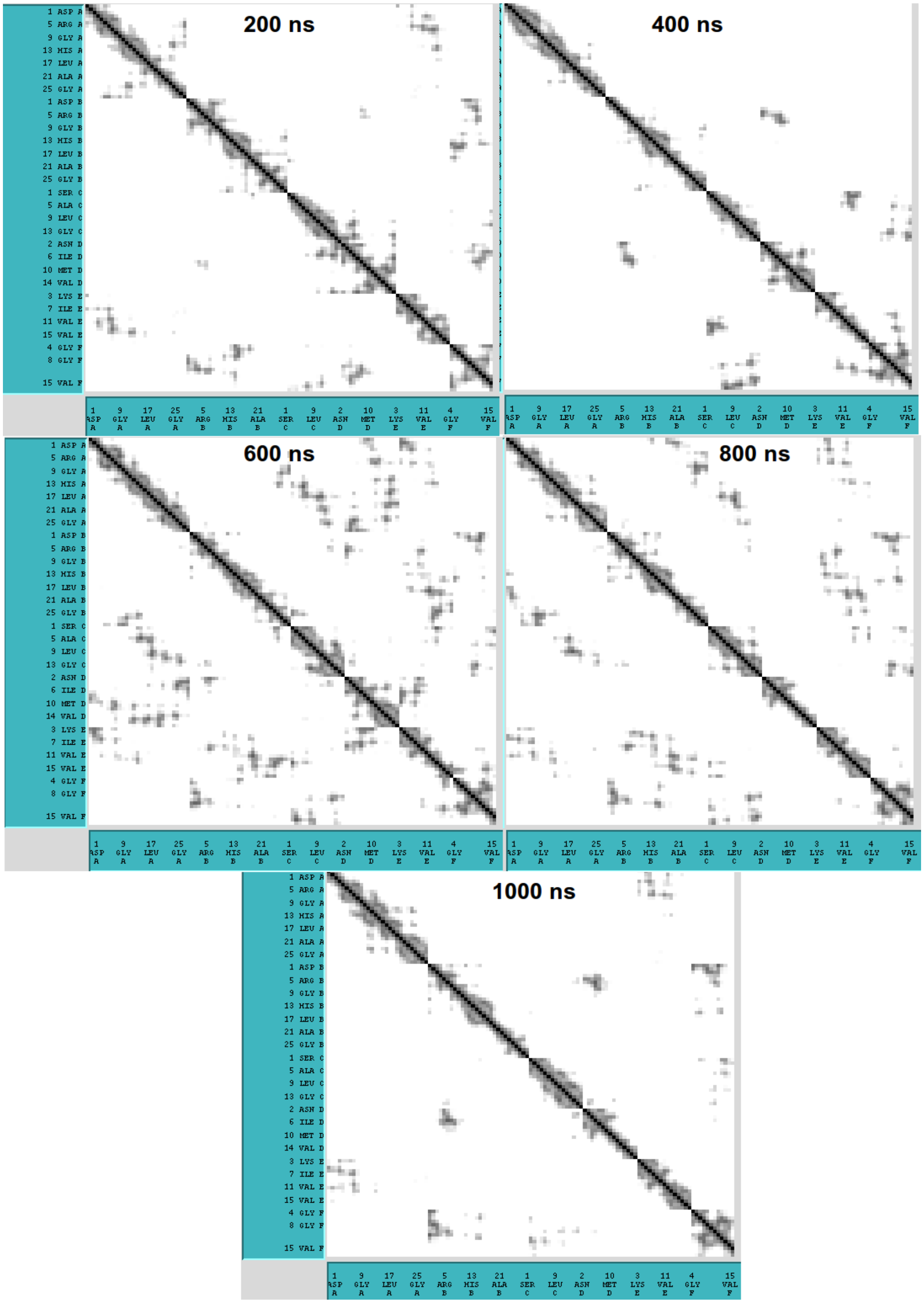

Figure S29. Contact maps computed during five 200 ns time intervals for mixed "AD" membrane containing both Aβ_{1-28} and Aβ_{26-40}. Letters A-F given on x and y axis represent different peptides.

Secondary structures of peptides

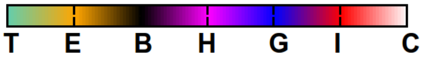

Figure S30. Secondary structure codes which are used in Figures S31-S44 according to the classification by Kabsch and Sander:[2] ”T” - turn (hydrogen bonded turn: 3,4 or 5 turn), ”E” - extended conformation (extended strand in parallel and/or anti-parallel β-sheet conformation with a minimal length of 2 residue), ”B” - isolated bridge (residue in isolated β-bridge; single pair β-sheet hydrogen bond formation), ”H” - α-helix (4-turn helix with a minimal length of 4 residues), ”G” - 3-10 helix (3_{10} helix with a minimal length of 3 residues), ”I” - π-helix (5-turn helix with the minimal length of 5 residues), ”C” - coil (none of listed earlier conformations).

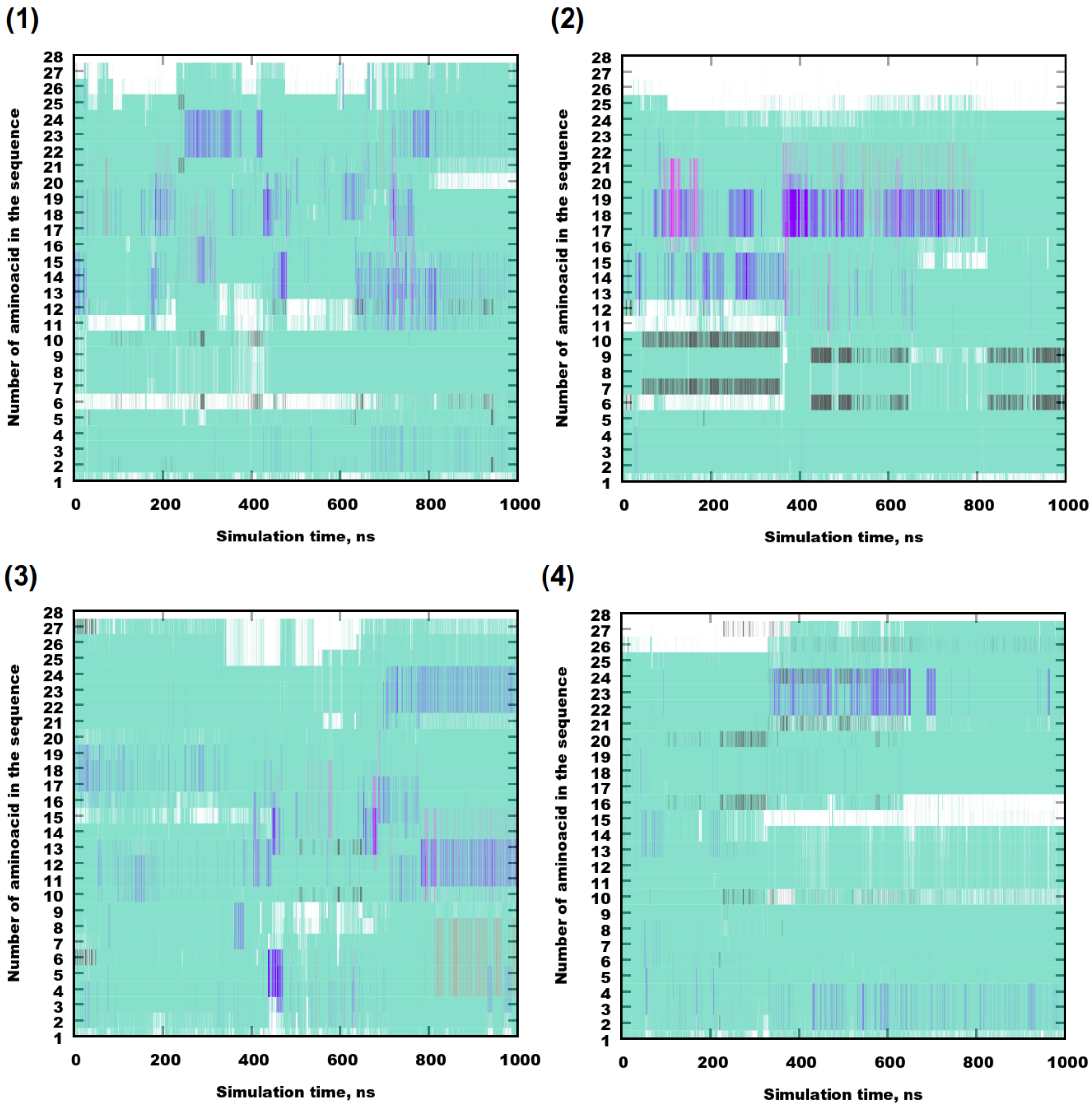

Figure S31. Secondary structure of Aβ_{1-28} peptides in 14:0-14:0 PC bilayer. Panels (1)-(4) correspond to different peptide molecules.

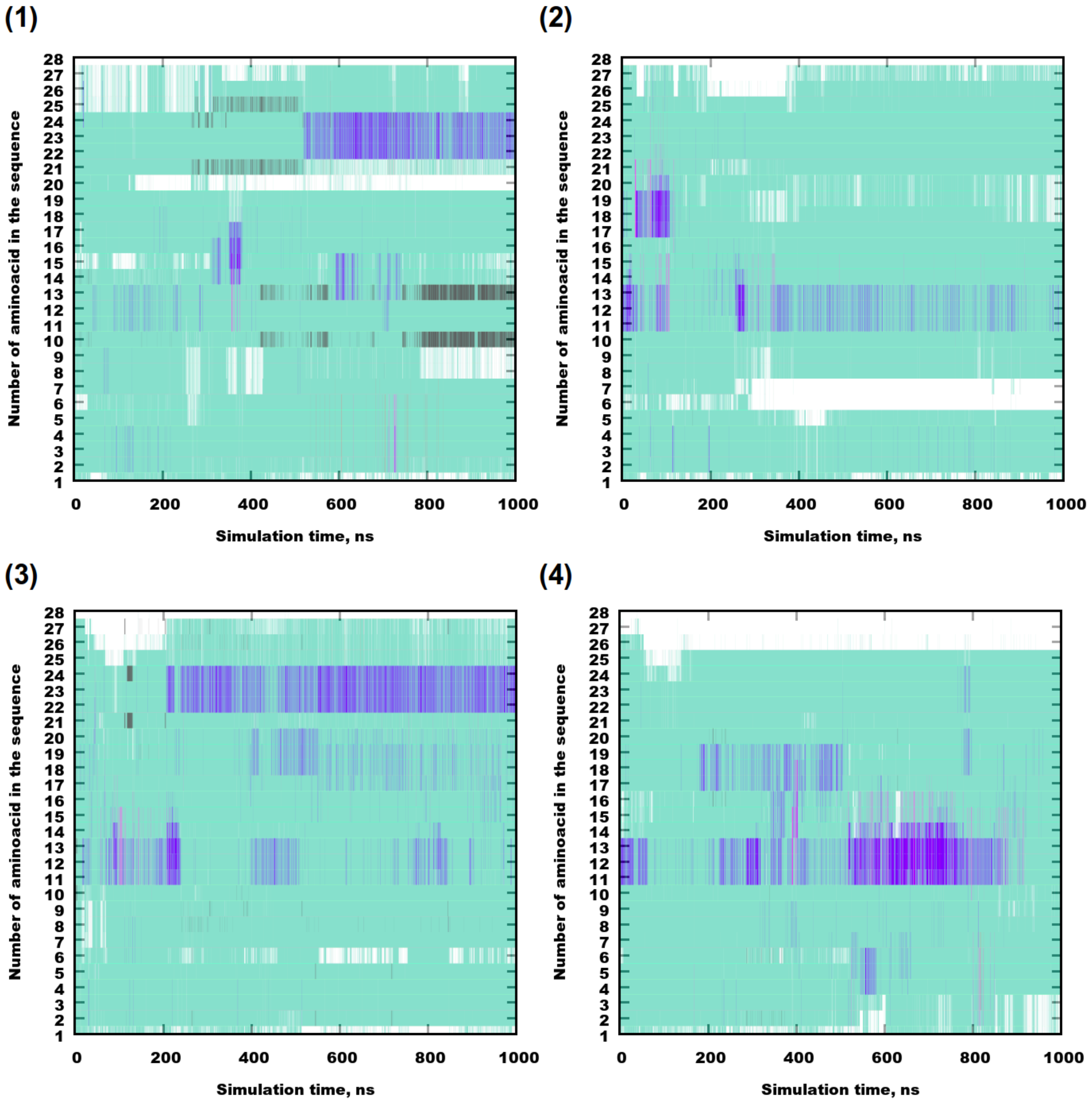

Figure S32. Secondary structure of Aβ_{1-28} peptides in 18:0 - 22:6 PC bilayer. Panels (1)-(4) correspond to different peptide molecules.

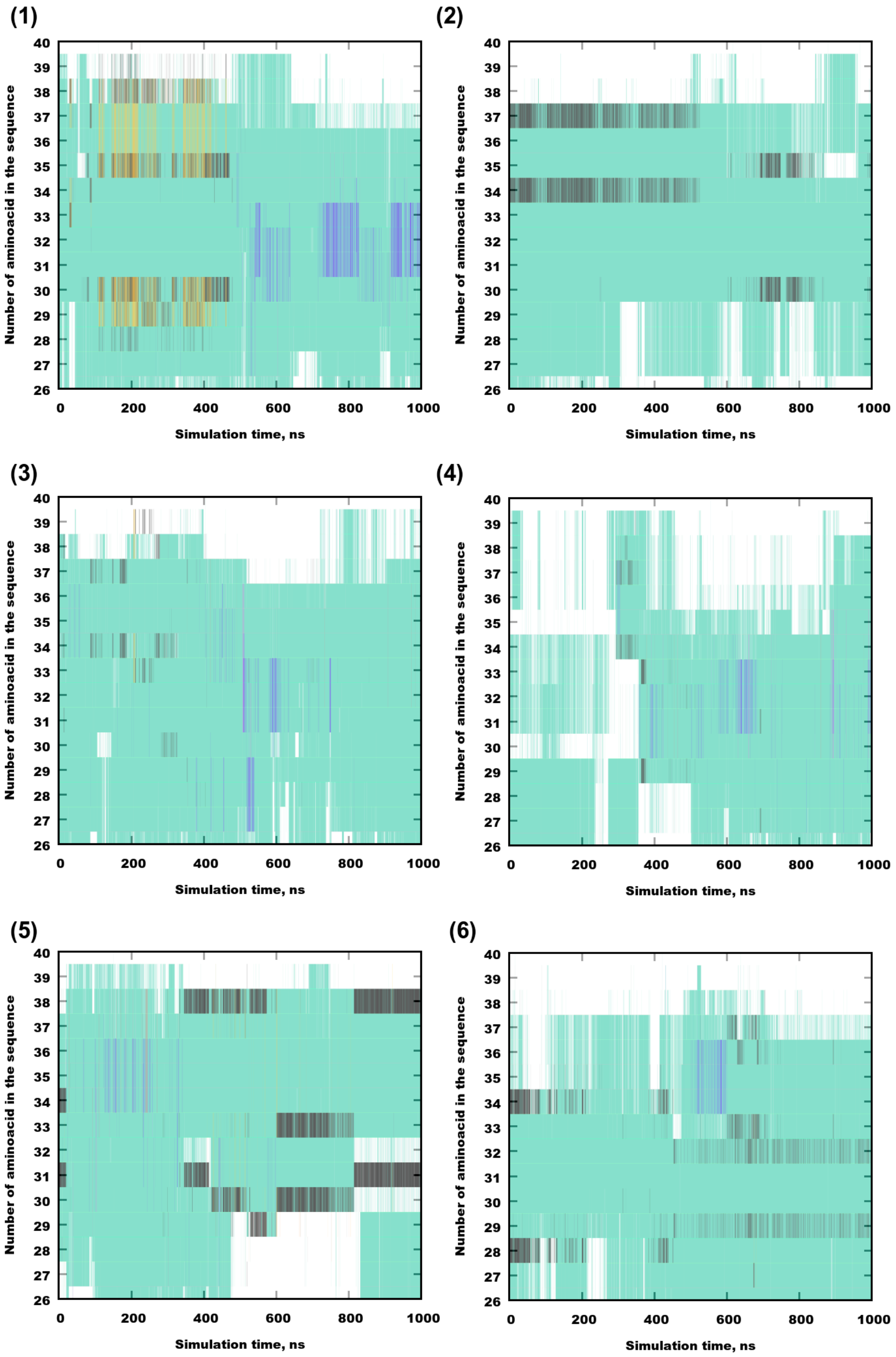

Figure S33. Secondary structure of Aβ_{26-40} peptides in 14:0 - 14:0 PC bilayer. Panels (1)-(6) correspond to different peptide molecules.

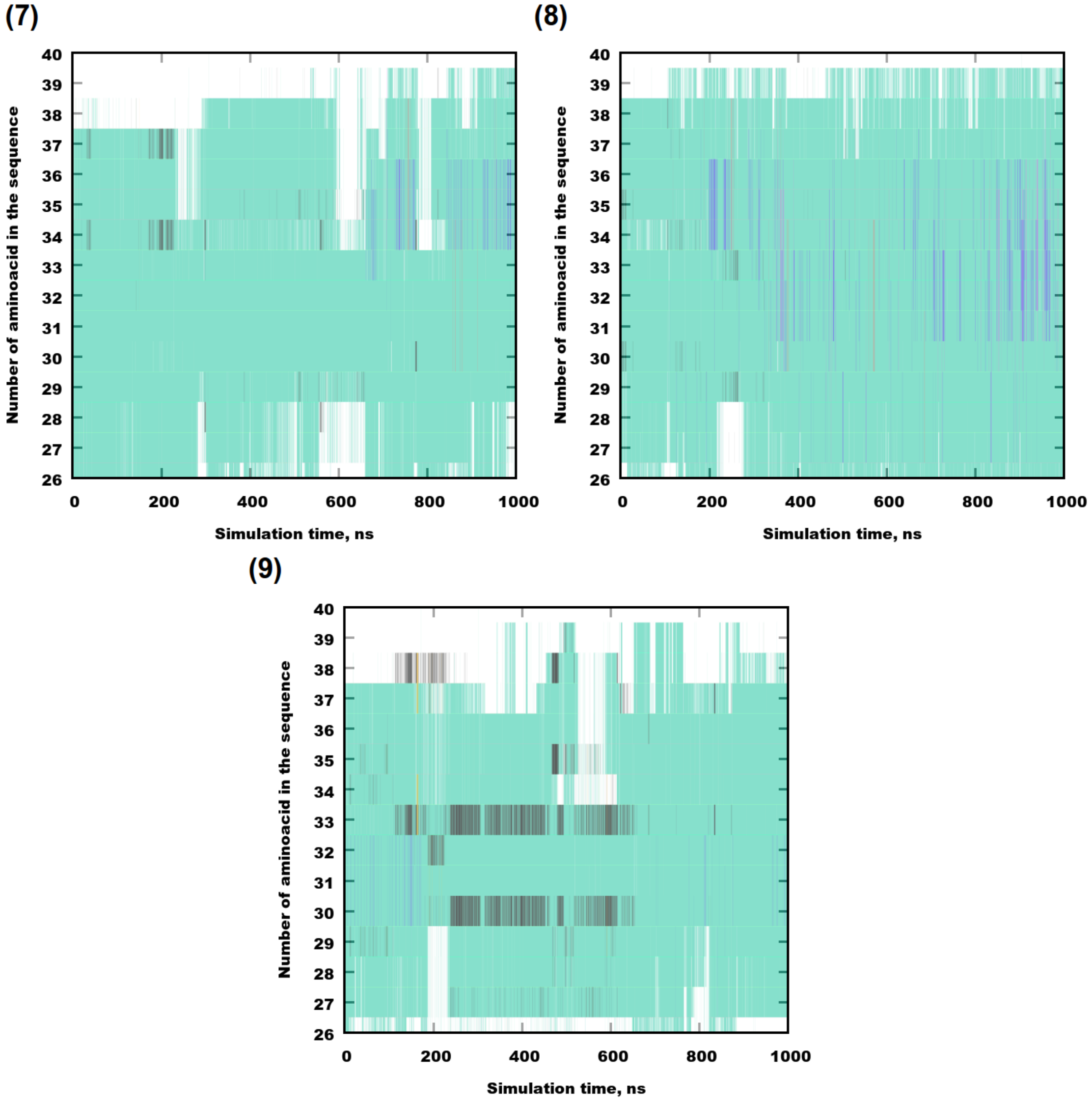

Figure S33(cont). Secondary structure of Aβ_{26-40} peptides in 14:0 - 14:0 PC bilayer. Panels (7)-(9) correspond to different peptides.

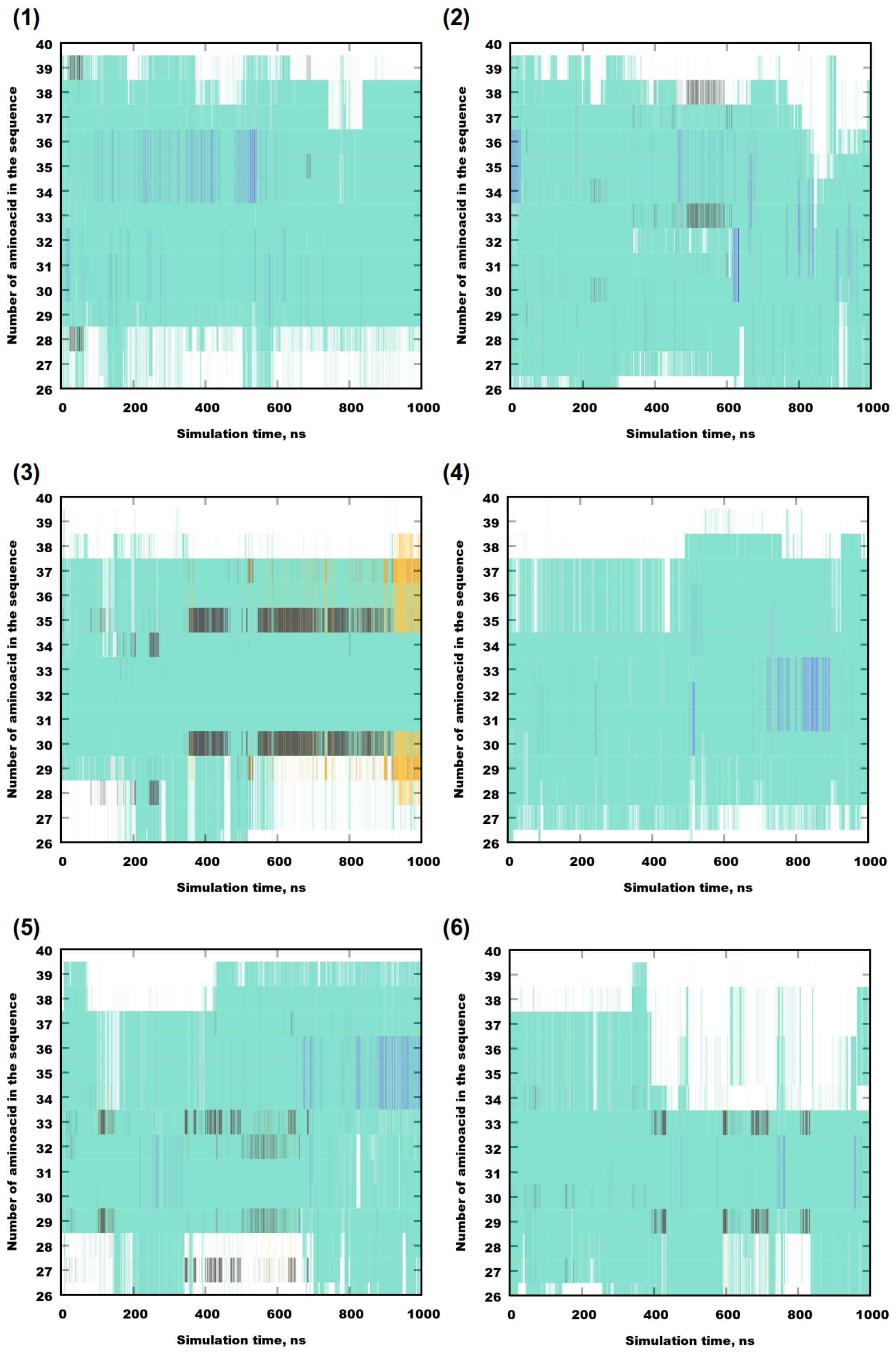

Figure S34. Secondary structure of Aβ_{26-40} peptides in 18:0 - 22:6 PC bilayer. Panels (1)-(6) correspond to different peptides.

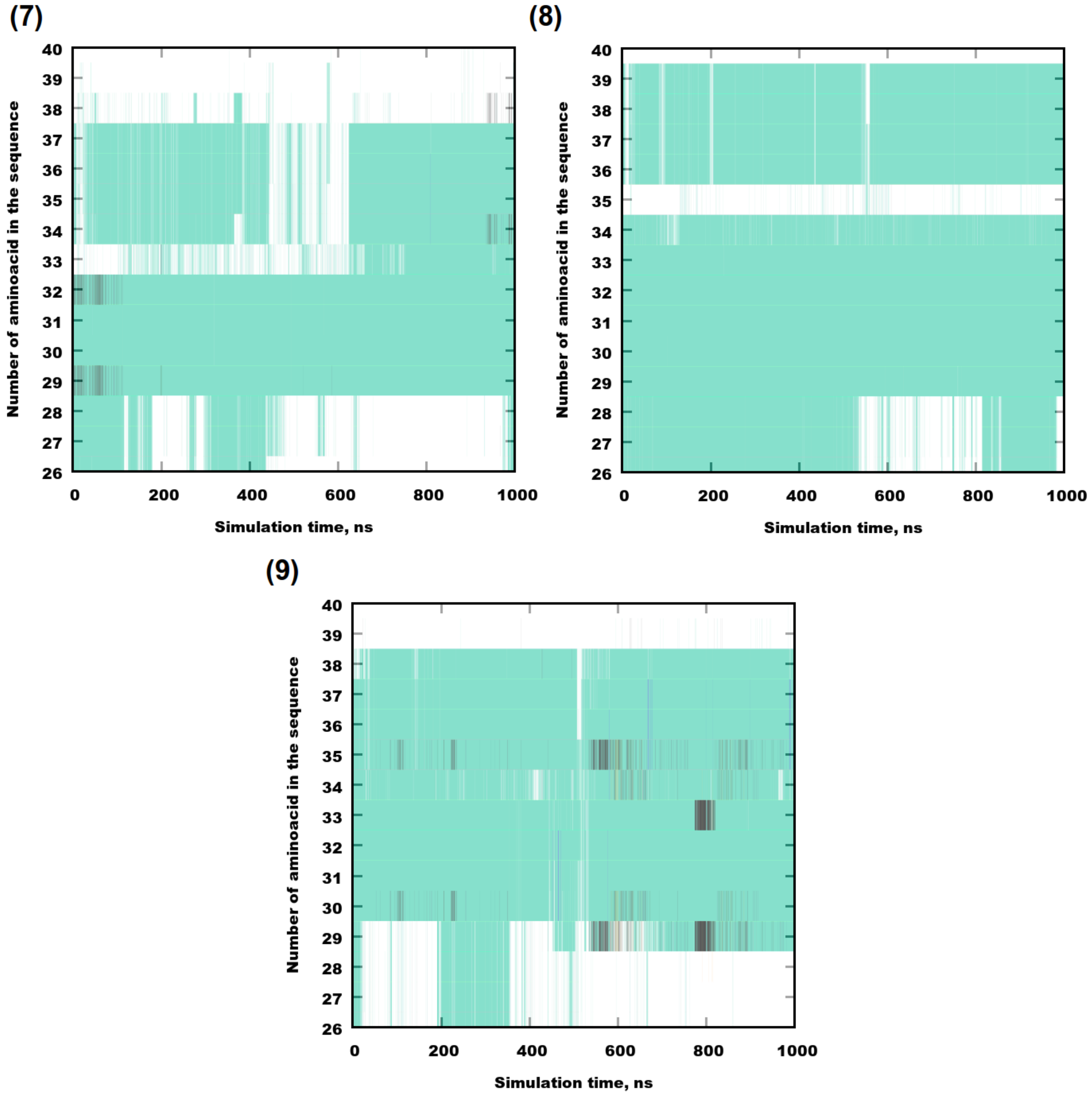

Figure S34a. Secondary structure of Aβ_{26-40} peptides in a mono-component 18:0 - 22:6 PC bilayer. Panels (7)-(9) correspond to different peptides.

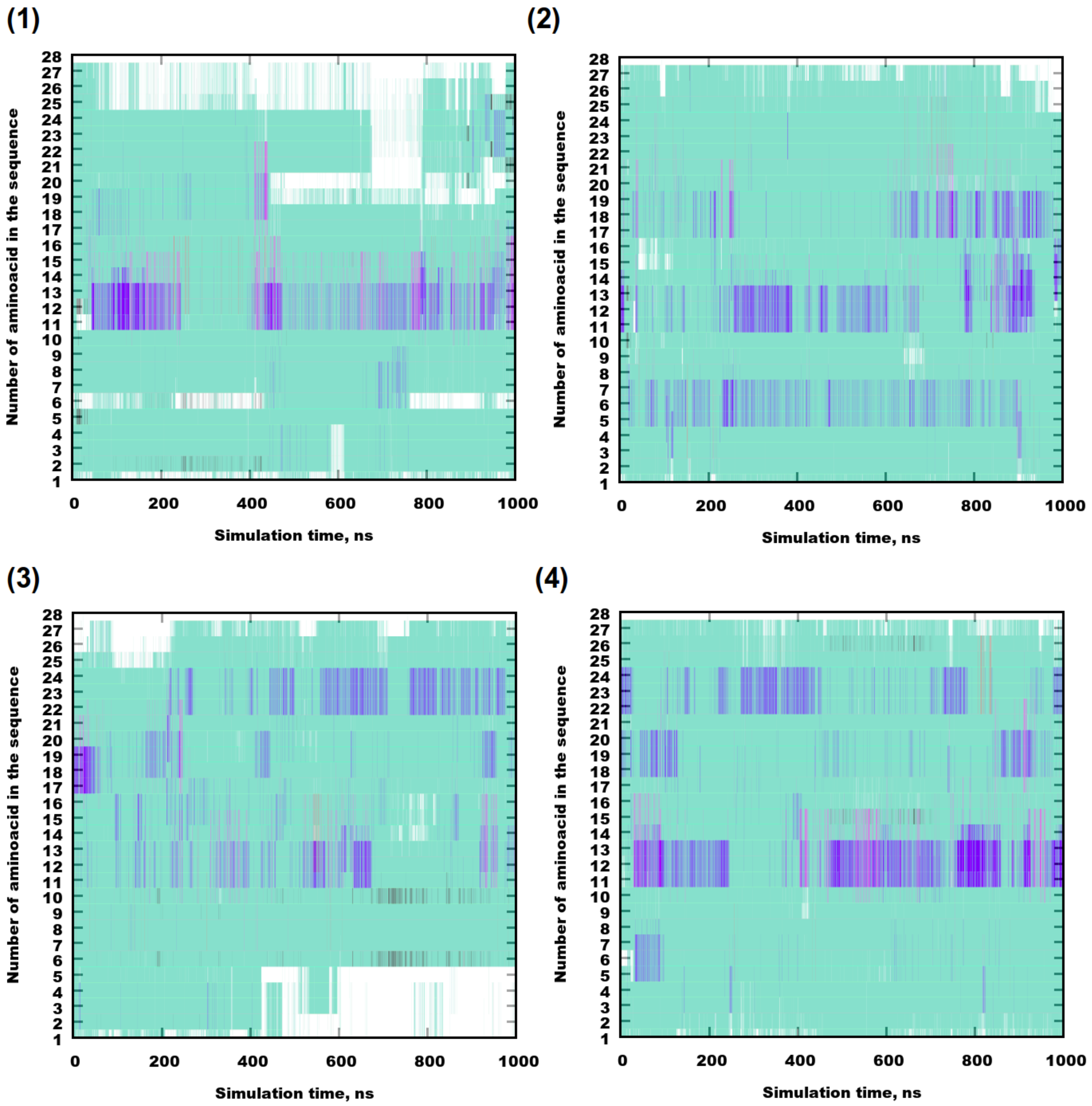

Figure S35. Secondary structure of peptides in mixed "normal" bilayer containing $A\beta_{1-28}$. Panels (1)-(4) correspond to different peptide molecules.

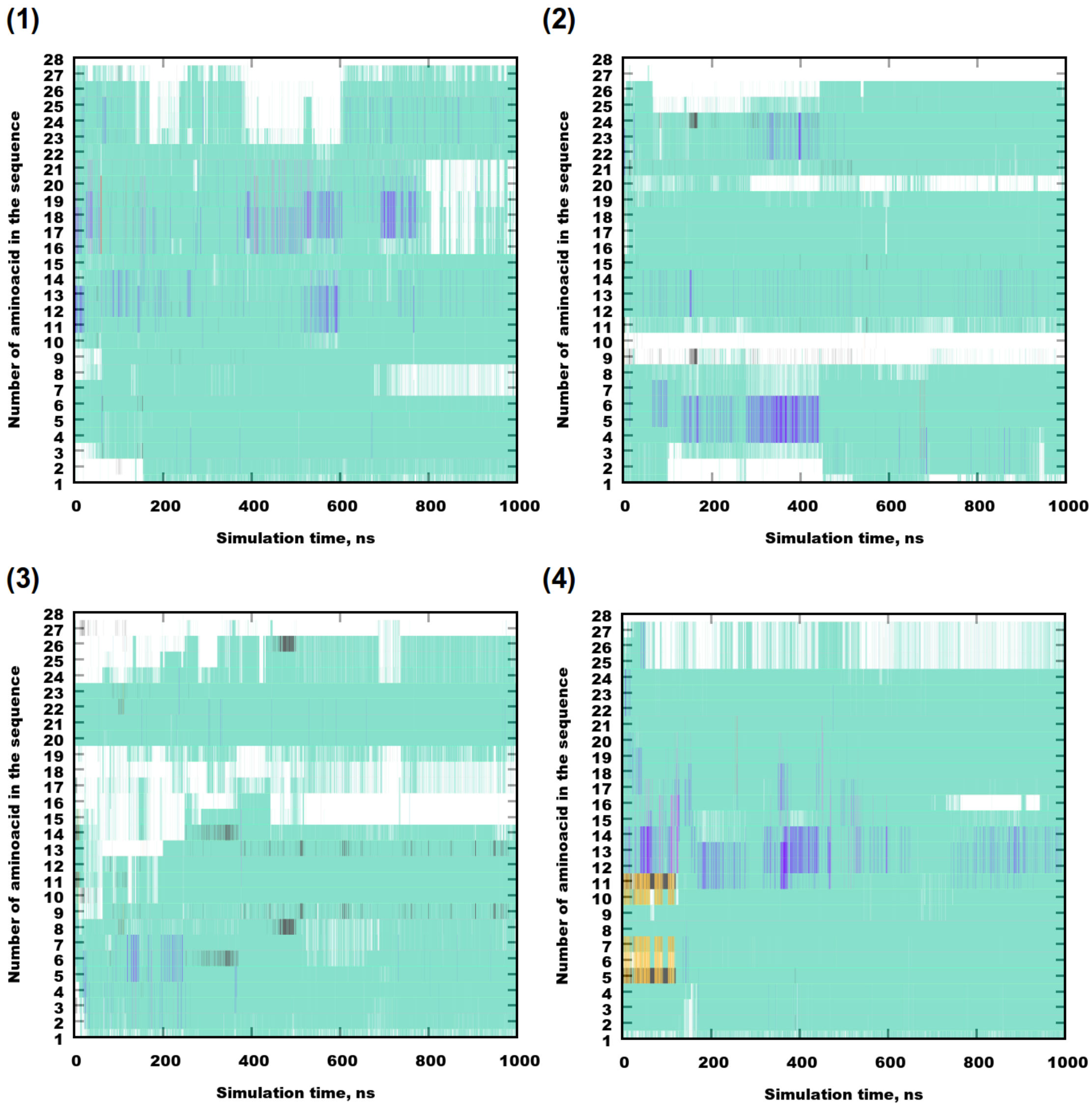

Figure S36. Secondary structure of peptides in mixed "AD" bilayer containing Aβ_{1-28}. Panels (1)-(4) correspond to different peptide molecules.

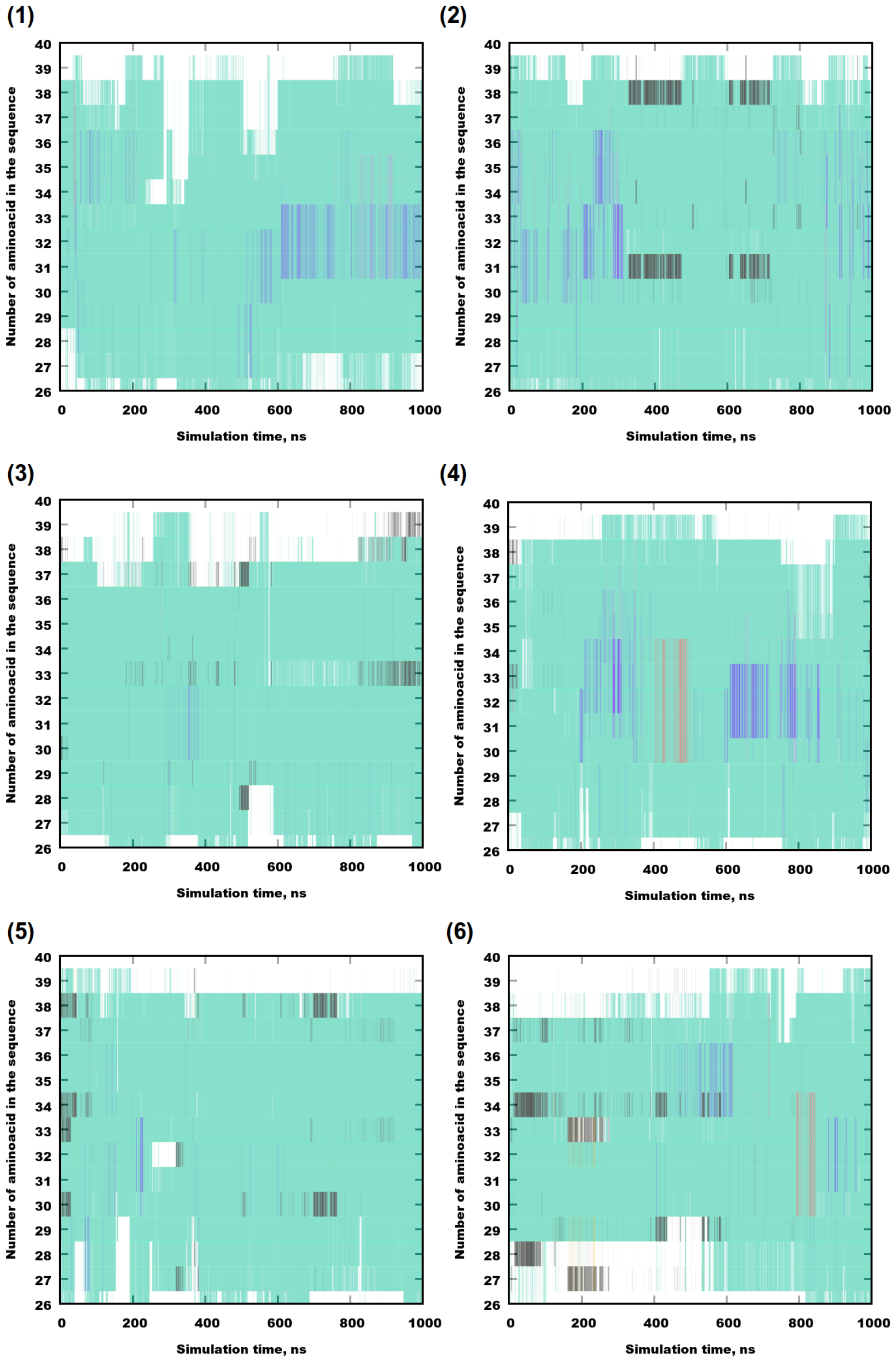

Figure S37. Secondary structure of peptides in mixed "normal" bilayer containing Aβ_{26-40}. Numbers (1)-(6) correspond to different peptide molecules.

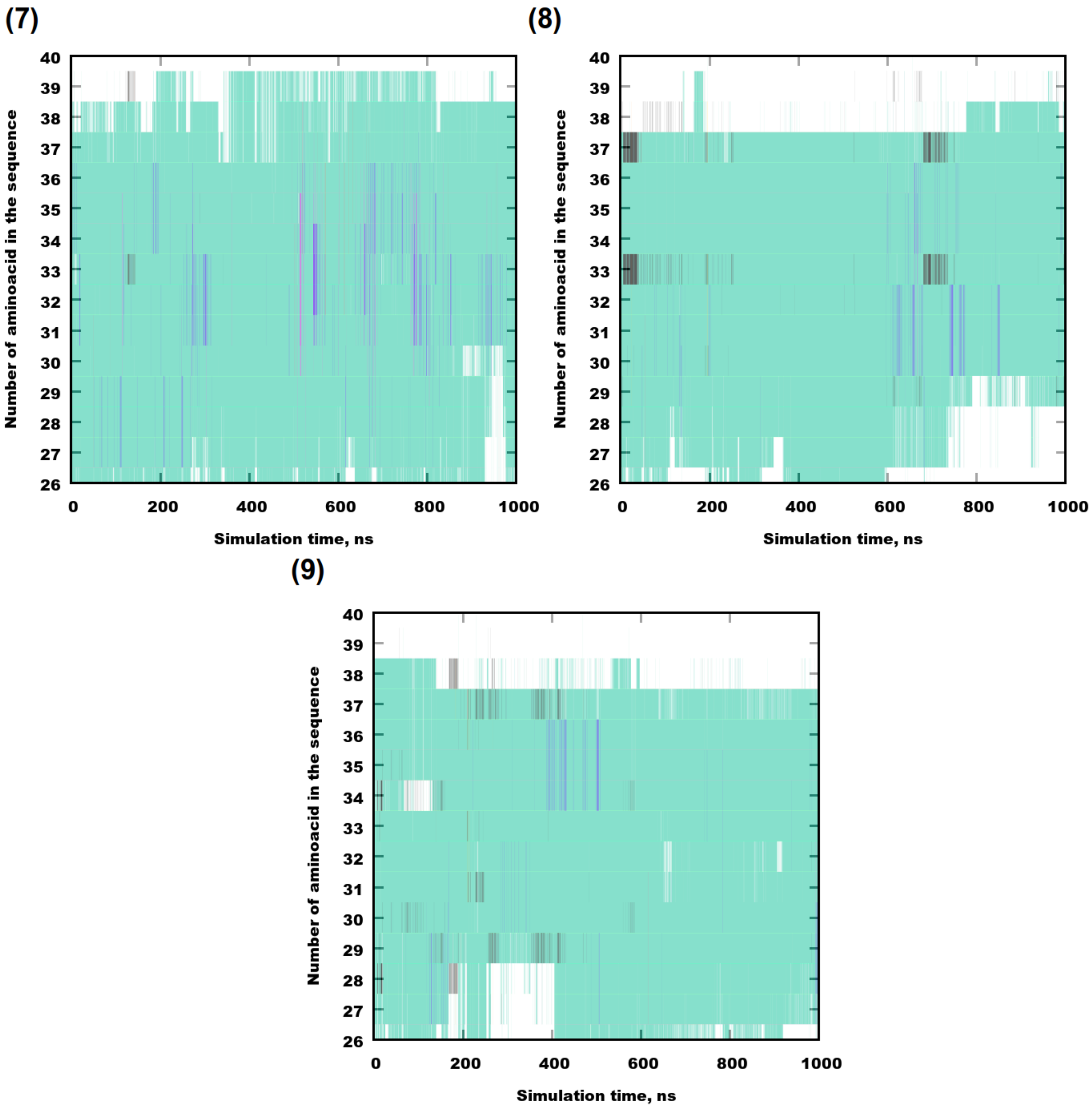

Figure S37 (cont). Secondary structure of peptides in mixed "normal" bilayer containing $A\beta_{26-40}$. Panels (7)-(9) correspond to different peptide molecules.

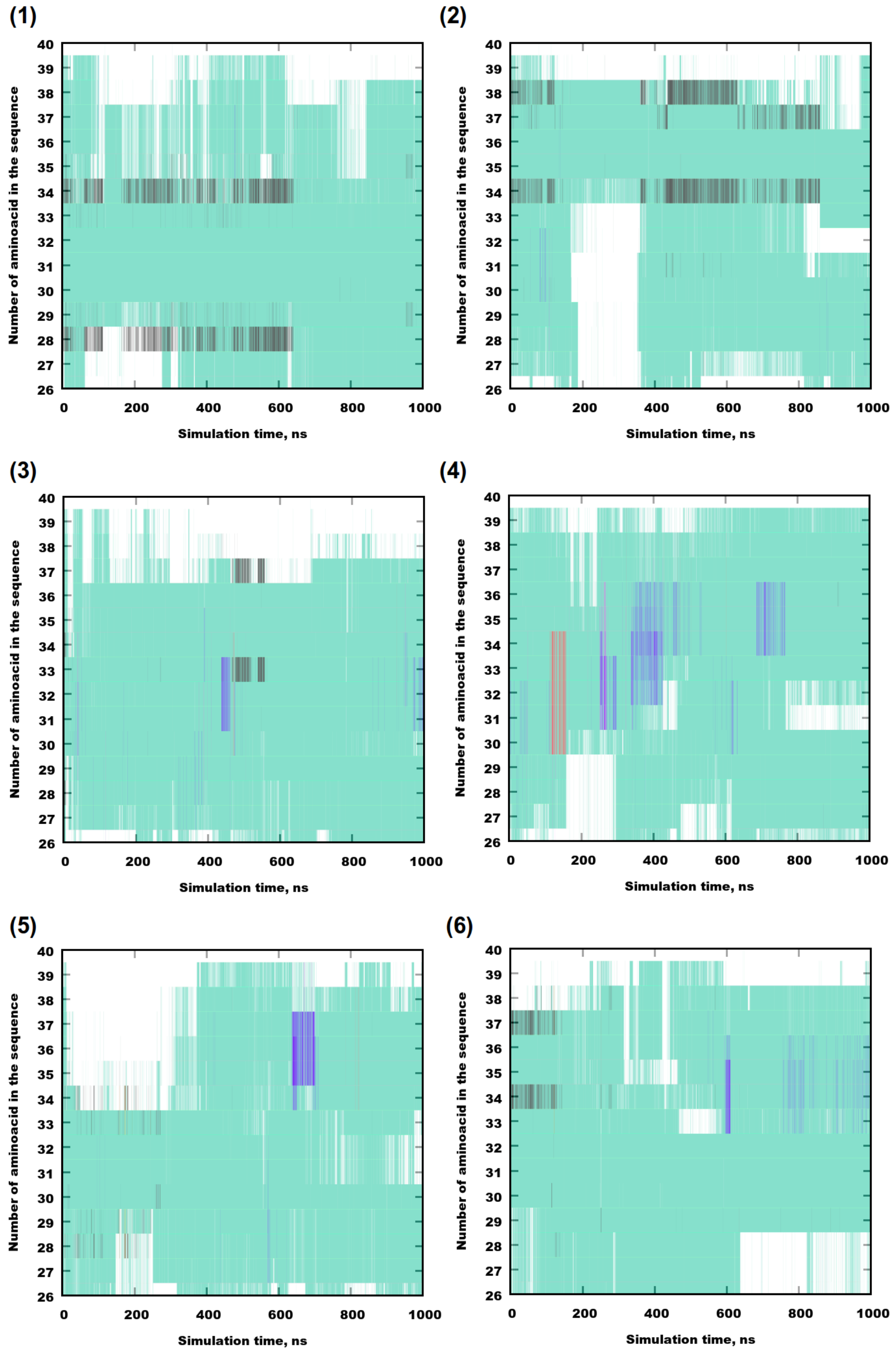

Figure S38. Secondary structure of peptides in mixed "AD" bilayer containing Aβ_{26-40}. Panels (1)-(6) correspond to different peptide molecules.

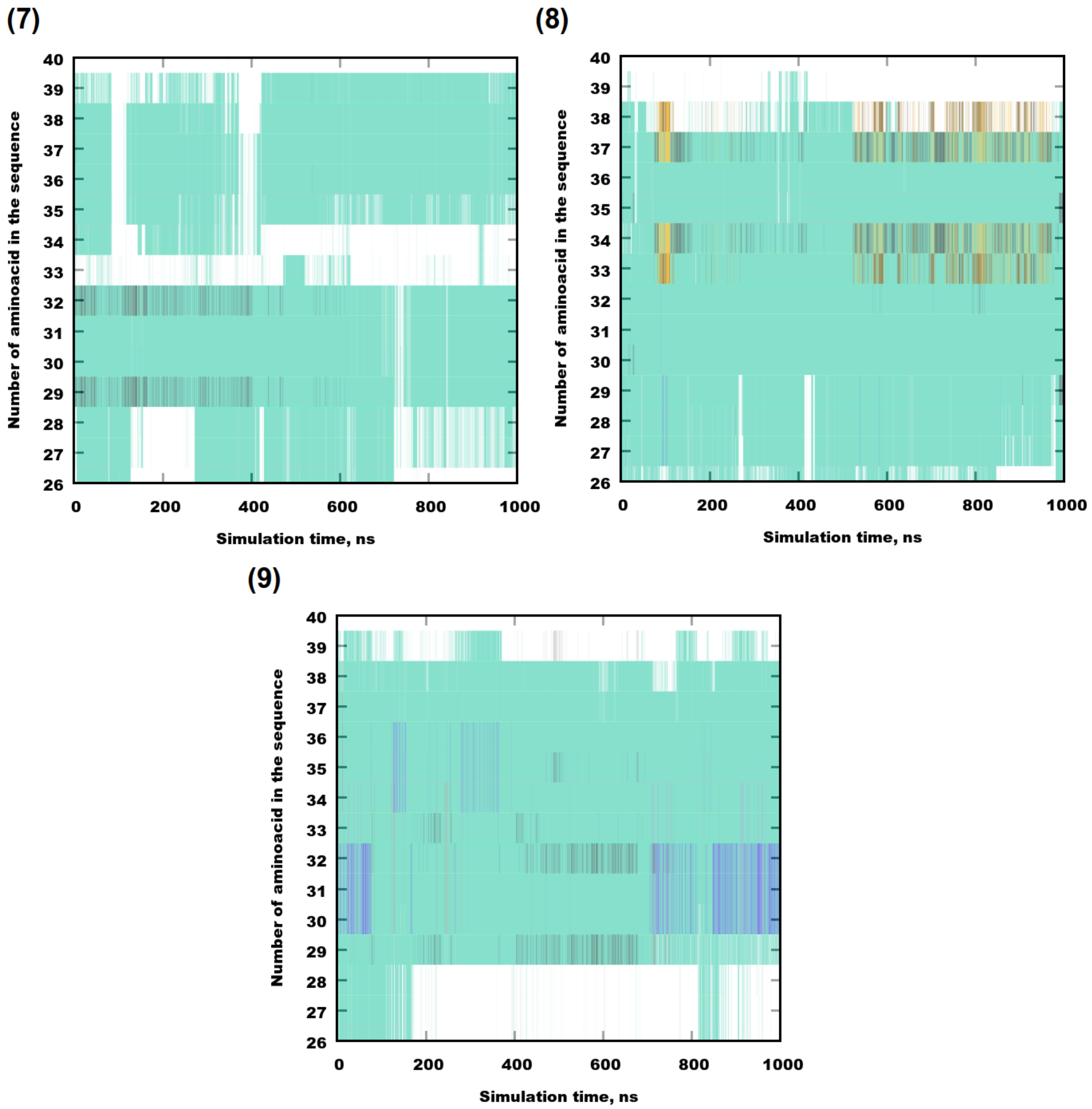

Figure S38 (cont). Secondary structure of peptides in mixed "AD" bilayer containing Aβ_{26-40}. Panels (7)-(9) correspond to different peptide molecules.

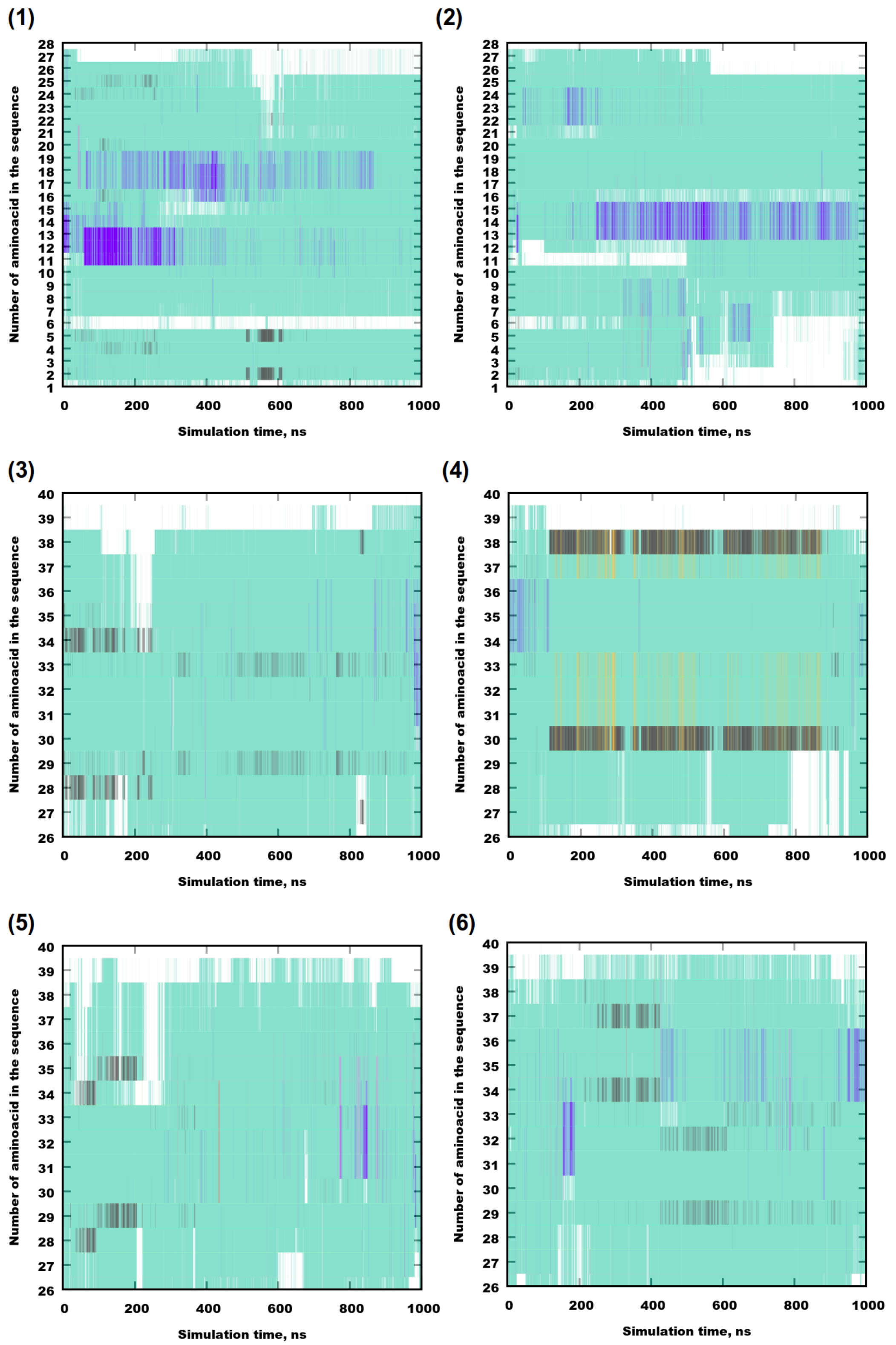

Figure S39. Secondary structure of peptides in mixed "normal" bilayer containing Aβ_{1-28} and Aβ_{26-40}. Panels (1) and (2) correspond to different Aβ_{1-28} and (4)-(6) to different Aβ_{26-40} peptide molecules.

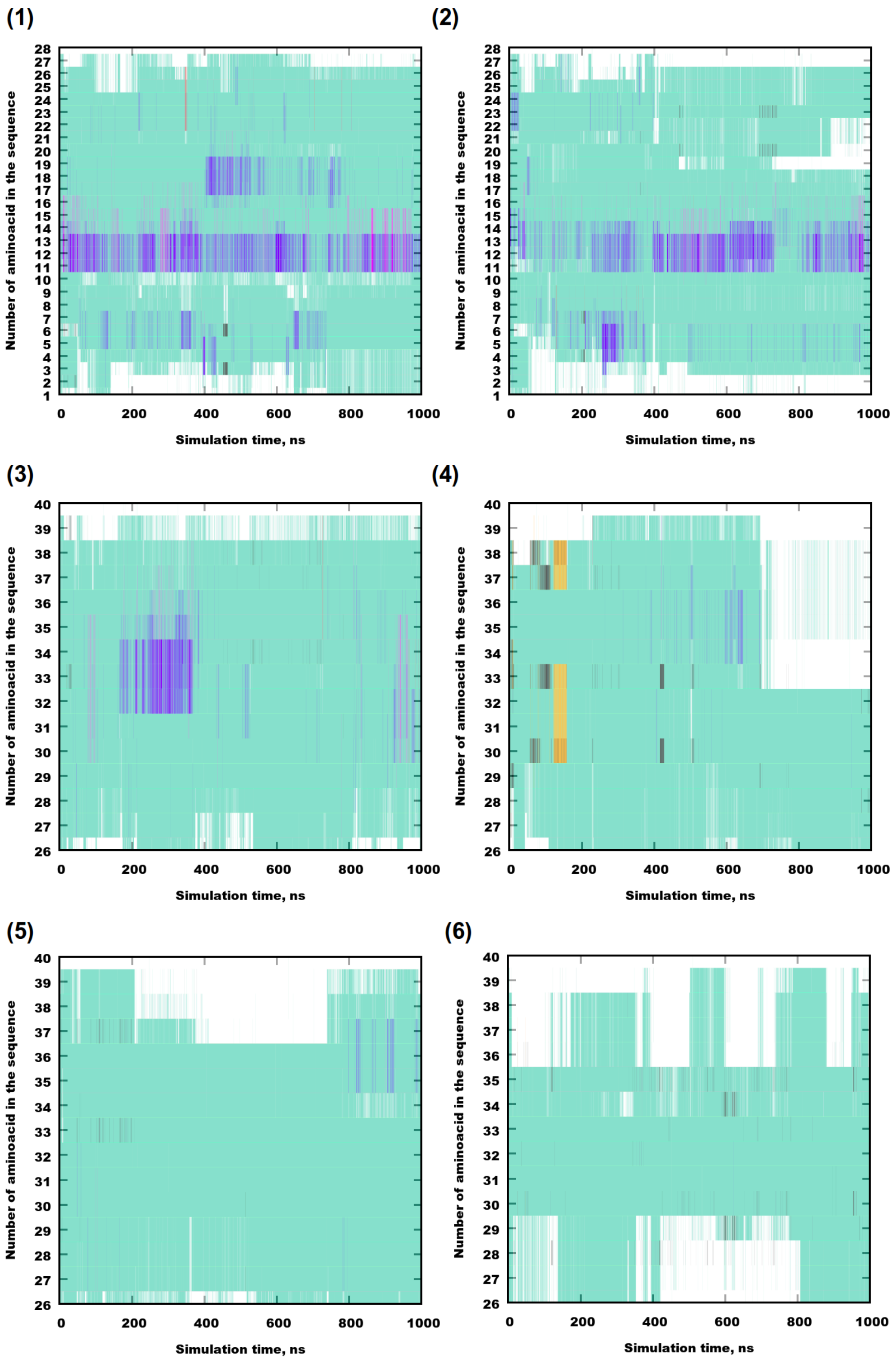

Figure S40.Secondary structure of peptides in mixed "AD" bilayer containing Aβ_{1-28} and Aβ_{26-40}. Panels (1) and (2) correspond to different Aβ_{1-28} and (4)-(6) to different Aβ_{26-40} peptide molecules.

References

(1) Humphrey, W.; Dalke, A.; Schulten, K. VMD: Visual Molecular Dynamics. *J. Mol. Graphics* **1996**, *14*, 33 – 38.

(2) Kabsch, W.; Sander, C. Dictionary of Protein Secondary Structure: Pattern Recognintion of Hydrogen-Bonded and Geometrical Features. *Biopolymers* **1983**, *22*, 2577 – 2637.
